# A novel, expert-endorsed, neurocognitive digital assessment tool for addictive disorders: development and validation study

**DOI:** 10.2196/44414

**Published:** 2023-08-25

**Authors:** Rico S. C. Lee, Lucy Albertella, Erynn Christensen, Chao Suo, Rebecca A. Segrave, Maja Brydevall, Rebecca Kirkham, Chang Liu, Leonardo F. Fontenelle, Samuel R. Chamberlain, Kristian Rotaru, Murat Yücel

**Affiliations:** 1 BrainPark, Turner Institute for Brain and Mental Health, Monash University, Melbourne, Australia; 2 Melbourne School of Psychological Sciences, University of Melbourne, Melbourne, Australia; 3 Obsessive, Compulsive, and Anxiety Spectrum Research Program, Institute of Psychiatry, Federal University of Rio de Janeiro (UFRJ); 4 D’Or Institute for Research and Education, Rio de Janeiro, Brazil; 5 Department of Psychiatry, University of Southampton, Southampton, United Kingdom; and Southern Health NHS Foundation Trust, Southampton, UK; 6 Monash Business School, Monash University, Melbourne, Australia

## Abstract

**Background:**

Many people with harmful addictive behaviours may not meet formal diagnostic thresholds for a disorder. A dimensional approach, by contrast, including clinical and community samples is potentially key to early detection, prevention, and intervention. Importantly, while neurocognitive dysfunction underpins addictive behaviours, established assessment tools for neurocognitive assessment are lengthy and unengaging, difficult to administer at scale, and not suited to clinical or community needs. The BrainPAC Project sought to develop and validate an engaging and user-friendly digital assessment tool, purpose-built to comprehensively assess the main consensus-driven constructs underpinning addictive behaviors.

**Objective:**

To psychometrically validate a gamified battery of consensus-based neurocognitive tasks against standard laboratory paradigms, ascertain test-retest reliability, and determine their sensitivity to addictive behaviors (eg, alcohol use) and other risk factors (eg, trait impulsivity).

**Methods:**

Gold standard laboratory paradigms were selected to measure key neurocognitive constructs (Balloon Analogue Risk Task, BART; Stop Signal Task, SST; Delay Discounting Task, DDT; Value-Modulated Attentional Capture task, VMAC; Sequential Decision-Making Task, SDT), as endorsed by an international panel of addiction experts; namely, response selection/inhibition, reward valuation, action selection, reward learning, expectancy/reward prediction error, habit, and compulsivity. Working with game developers, BrainPAC tasks were developed and validated in three successive cohorts (total N = 600) and a separate test-retest cohort (N = 50) via Mechanical Turk using a cross-sectional design.

**Results:**

BrainPAC tasks were significantly correlated with the original laboratory paradigms on most metrics (r = 0.18 to 0.63, *P* < 0.05). With the exception of DDT k function and VMAC total points, all other task metrics across the five tasks did not differ between the gamified and non-gamified versions (*P* > 0.05). Four out of five tasks demonstrated adequate to excellent test-retest reliability (ICC = 0.72 to 0.91, *P* < .001; except SDT). Gamified metrics were significantly associated with addictive behaviours on behavioural inventories, though largely independent of trait-based scales known to predict addiction risk.

**Conclusions:**

A purpose-built battery of digital gamified tasks is sufficiently valid for the scalable assessment of key neurocognitive processes underpinning addictive behaviours. This validation provides evidence that a novel approach, purported to enhance task engagement, in the assessment of addiction-related neurocognition is feasible and empirically defensible. These findings have significant implications for risk detection and the successful deployment of next-generation assessment tools for substance (mis)use and other mental disorders characterised by neurocognitive anomalies related to motivation and self-regulation. Future development and validation of the BrainPAC tool should consider further enhancing convergence with established measures, as well as collecting population-representative data for use clinically as normative comparisons.

## Introduction

Addiction is defined as a chronic, relapsing disorder characterized by compulsive substance seeking, or other compulsive behaviours (eg, problem gambling), and is associated with an average loss of 14 years in life expectancy [1]. A key mechanism impacting addiction risk is cost-benefit decision-making ability [2]. The neurocognitive processes underpinning choice behaviors are complex and dynamic with distinct changes corresponding to differing stages of the addiction cycle [eg, transition from impulsivity to compulsivity; 3]. Despite our decades-old understanding of the cognitive mechanisms central to the development and maintenance of addiction – predominantly from pre-clinical research [4], but increasingly in the clinical neurosciences [5] – there is currently no purpose-built tool that comprehensively indexes key neurocognitive functions for people on the addiction spectrum. A comprehensive tool has the potential to facilitate early risk detection and monitoring in addiction, as well as to identify targets for early intervention, and measure potential cognitive effects of interventions. Additionally, there is an imperative for approaches that are more engaging than laboratory or pen-and-paper clinical tasks (eg, digital, user-friendly, gamified), given neurocognitive testing is particularly vulnerable to poor effort, especially when unsupervised. There is also a significant need for scalable, potentially ‘self-administered’ assessment technologies accessible to more people who may have limited access to traditional clinical services [6–9]. As such, an engaging and scalable (ie, mobile health or mHealth) tool designed to validly and sensitively measure neurocognitive and motivational risk to addictive behaviors is critical to meeting this unmet demand.

Current neurocognitive batteries are limited by their focus on functions that are not core to addictions. These functions were typically selected from neuropsychological tests intended for use in brain injury and neurological disorders, such as processing speed, attention span, episodic memory, and nonverbal reasoning, amongst others [10, 11]. Despite some being associated with risk for certain substance use disorders [eg, working memory and alcohol addiction; 12], they are not broadly thought to be mechanistically and causally linked to the perpetuation of substance and behavioral addictions, and as such cannot inform risk detection. That is, current approaches focus on problematic addictive behaviors (eg, alcohol misuse) and its neurocognitive *sequelae* (eg, memory impairment), rather than the underlying motivational or top-down neurocognitive, *antecedent* processes known to drive those behaviors [eg, inhibitory dyscontrol; 9].

By contrast, neurocognitive functions subserved by the neurocircuitries of motivation and self-regulation, which determine the risk of developing maladaptive ‘approach behaviours’ [7], are mechanistically linked to addictive behaviors. Guided by the NIMH-RDoC, a three-round international DELPHI consensus study of world-leading addiction experts identified key constructs mechanistically implicated in addiction-related outcomes; namely five *positive valence* constructs (reward valuation, expectancy/reward prediction error, action selection, reward learning, habit), a *cognitive control* construct (response selection/inhibition), and a non-RDOC expert-initiated construct [compulsivity; 13]. This is corroborated by the existing addiction neuroscience literature that has established key risk mechanisms linked to these functions. For example, the preference or the reward valuation of a smaller, immediate reward over a larger, later reward is a significant risk factor across a range of addictive behaviors [14, 15]. Despite our wealth of knowledge, a key barrier remains - that is, there is currently no psychometrically validated, consensus-driven neurocognitive battery to holistically assess these constructs. Narrow, single-construct approaches are insufficient given the significant overlap between relevant risk domains, which a comprehensive tool can help disentangle mechanisms (ie, what are unique risk factors).

**Fig. 1:**
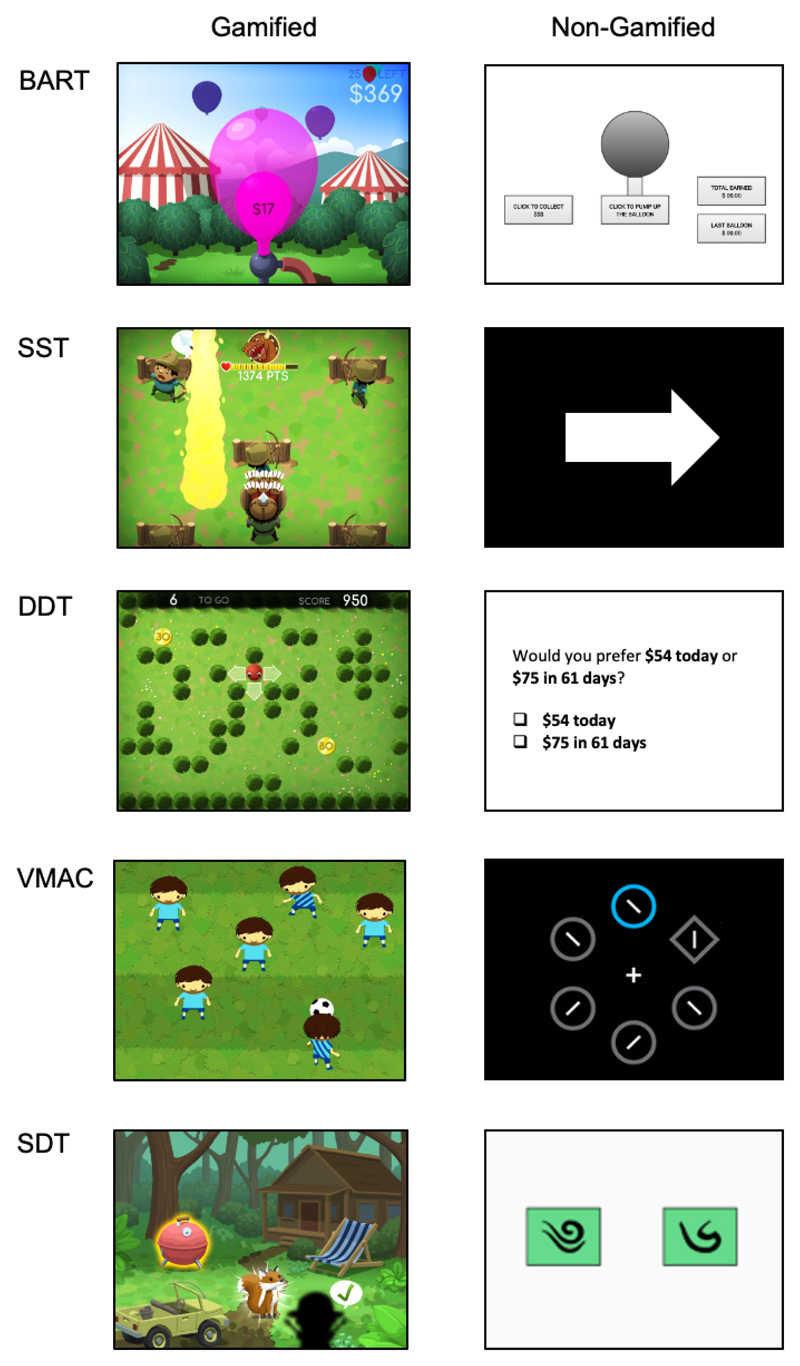
The gamified *BrainPAC* tasks compared to the non-gamified paradigms.

To this end, the BrainPark Assessment of Cognition (*BrainPAC*) digital assessment battery (Fig. 1) was developed. Given that accurate neurocognitive assessment relies on people giving strong and enduring effort to the task at hand [16], ‘gamification’ [ie, the re-design of tasks to include game elements; 17], was used with the goal of addressing the potential motivational challenges that stem from unsupervised testing environments and are inherent to app-based assessment tools. Gamification was conducted carefully and iteratively between neuropsychologists (RSCL, RS, MY), industry partners, and community focus groups over regular workshops to ensure that paradigms continue to retain the requisite neurocognitive elements and, as such, remain valid and reliable, whilst being engaging to end-users. Of relevance, there is evidence that gamification can introduce additional cognitive demands [18] and efforts were made at all stages of development to minimise these additional demands. *BrainPAC* therefore represents a shift in how we approach traditional cognitive assessment in that user-friendliness, engagement and expert-endorsement are driving principles in task development. Given the emerging evidence behind the gamified approach, BrainPAC ultimately serves as a proof-of-principle for future mHealth innovations.

Five cognitive paradigms were developed into gamified tasks: the Balloon Analogue Risk Task (BART) to index action selection; Stop Signal Task (SST) to index response selection/inhibition; Delay Discounting Task (DDT) to index reward valuation; Value-Modulated Attentional Capture (VMAC) reversal task to index reward learning and compulsivity-related cognitive flexibility; and Sequential Decision-Making Task (SDT) to index reward learning and expectancy/reward prediction error. In addition to neurocognition, the literature also documents a consistent relationship between trait-based factors (eg, trait impulsivity and trait compulsivity) and addiction risk [3, 19–24]. Therefore, the *BrainPAC* tool goes beyond cognition to include gold standard, structured trait (eg, impulsivity, compulsivity) and behavioral (eg, alcohol use) structured scales [eg, 13, 25, 26, 27], as well as a personalised feedback system that tracks and presents changes over time. However, these latter components are beyond the scope of this current Stage 1 validation and only the neurocognitive validation data will be presented herein. Stage 2 and 3 of the BrainPAC validation project will address the other components of the BrainPAC tool. In total, the five gamified tasks take approximately sixty minutes to complete.

We sought to empirically validate the gamified BrainPAC tasks alongside the original laboratory paradigms, the recommended gold standard approach [17]. We hypothesised that BrainPAC task metrics (ie, sub-scores from each measure, such as Go Reaction Time on the SST) would not significantly differ from the original laboratory-based task metrics. Further, we predicted that gamified metrics would yield large correlations with the original metrics and would demonstrate expected divergence or small associations with trait-based measures (ie, impulsivity, compulsivity), as well as small to moderate corrections with common addictive behaviors (eg, alcohol use). We chose to focus here on general community samples irrespective of clinical or diagnostic thresholds.

## Methods

### Recruitment

Six-hundred-and-fifty participants were recruited from Mechanical Turk [28]. Mechanical Turk is an online crowdsourcing platform that facilitates rapid recruitment and has been shown to yield more demographically diverse samples than traditional samples and other internet samples [29–31]. Indeed, Mechanical Turk samples tend to include individuals with greater mental health and addiction burden [29–31]. Importantly, the platform indexes users on the quality of their work by an ‘approval rating’ (ie, percentage of jobs completed satisfactorily), with data showing that restricting recruitment to users with >95% approval rating yields high-quality data for research [32]. Data quality in the current study was further enhanced through implementing validity questions, screening for reduced effort, as well as the exclusion of data from individuals who completed tasks within implausible timeframes. All data collected was non-identifiable and stored on password-protected local servers with the investigators having sole access.

Inclusion criteria were age of 18-55 years of age and fluency in English. This study was approved by the Monash University Human Research Ethics Committee (approval ID: 8239) and all participants gave informed, written consent prior to participation. Participants were reimbursed US$1 for every 10 minutes of research participation.

### Study Design

Participants were recruited in three consecutive cohorts for task validation, which coincided with task development milestones dates. The first phase of recruitment occurred in August 2019 (BART and SST); the second (DDT and VMAC) and third (SDT) occurred in May 2020. We chose not to validate all tasks within a single sample to minimise fatigue effects within each cohort, since the combined length of tasks was very long as participants completed both the gamified and standard versions of each paradigm. This was done with the exception of the SDT, which is the longest of the five tasks, as we already had access to comparable data provided by authors from the non-gamified paradigms. The order that the gamified and standard versions were presented in each cohort was counterbalanced to minimise order effects. A separate sample was recruited to examine test-retest reliability, again with task order counterbalanced.

### Measures

#### Balloon Analogue Risk Task

The BART indexes action selection within the RDoC matrix. It measures the tendency to make risky decisions, and how an individual balances the potential for risk versus reward [33]. Risky decision-making on the BART is linked to real-world risk-taking, including unsafe driving [34], alcohol use [35], drug use [36], and risky sexual behavior [37].

In keeping with the standard paradigm, participants are presented with balloons on the gamified BART and offered the chance to earn money by sequential inflation decisions. Each balloon pump earns the player a potential monetary unit of reward. Once a (predetermined pseudorandomised) burst threshold is reached the balloon will explode and all earnings from that balloon are lost. Thus, each pump increases the risk of a loss, while at the same time increases the potential reward. We created a ‘pre-commitment’ version of the BART, where players have to decide the number of pumps they want to make (ie, risk) immediately upon presentation of each new balloon, rather than over an extended period of individual manual ‘pumps’ (ie, button presses) as per the traditional paradigm [38, 39]. This variant has been shown to be a more accurate reflection of risk-taking given the amount of risk a player is willing to make on burst balloons is predetermined, in contrast to the standard, manual ‘pump BART’ where an intended risk is lost on burst trials [39]. The task was designed so individuals can stretch the balloon to their desired size (potential monetary reward) by keeping a finger pressed on the left mouse key, before banking by letting go of the cursor. Three practice trials preceded 30 test trials. The mean burst point was set at 64 pumps (out of 128). The task takes approximately five minutes. Four key metrics were computed based on prior studies [39]: (total) bursts, mean (pre-committed) pumps, (total) money earned, and coefficient of variability.

#### Stop Signal Task

SST is one of the most commonly used response inhibition tasks [40, 41] and was chosen to measure the construct of response selection/inhibition. In this task, participants are asked to press a button (eg, left button) in response to a stimulus (eg, left arrow), and another (eg, right button) in response to another stimulus (eg, right arrow). This choice reaction time part is known as the ‘go’ trials. On a portion of trials, however, a ‘stop signal’ stimulus (eg, a coloured dot or a beep sound) will appear after a delay (known as the ‘stop signal delay’; SSD), requiring participants to inhibit an already initiated but latent go-trial ‘response’. The SSD is stair-cased (ie, difficulty-level decreases or increases according to performance) to ensure performance is kept close to 50% correct. A ‘stop signal reaction time’ (SSRT) is computed from the distribution of responses indexing the average time required to inhibit an initiated go response. Response inhibition on the SST has been associated with a range of addictive behaviours, including alcohol misuse, opioid dependence and problem gambling [6].

In the gamified SST, players engage in a battlefield game to replenish arrow supplies of team-mates on the battlefield. Players press left or right to move the character up the grid to restock arrows as quickly as they can when signalled by one of two archers. In a minority of trials (ie, 30%), the enemy dragon breathes fire on the battlefield, necessitating the player to withhold their response (ie, the ‘stop trials’). We incorporated a reward system to incentivise faster go responses using a points system (and reduce the chance of players ‘waiting’ for the stop signal as a strategy) as previously recommended by a panel of experts [42], whereby greater points were awarded when correct go responses were quicker (up to a maximum of 25 points per trial). A progress bar and sound effects were included to further enhance engagement. There were 10 practice and 150 test trials, with SSD starting at 200ms and stair-cased by 50ms. Participants were required to achieve 70% correct go responses before proceeding to test trials (or had to repeat the practice). The gamified SST tasks approximately 12 minutes. Here we calculated SSRT using both the mean and integration methods [42], as well as computing the mean go reaction time (Go RT).

#### Delay Discounting Task

The monetary choice questionnaire (MCQ) is the most commonly used DDT paradigm [43] and was chosen to index reward valuation, given temporal discounting is a key and the most well-understood component of this construct. DDT measures the propensity to seek a reward of lower value that is quicker to obtain, over seeking a larger reward that takes more time to acquire [44]. A bias toward instant gratification over long-term goals is characteristic of individuals who are considered impulsive, often contributing to problems such as substance abuse [43], smoking [45], gambling [46] and obesity [47]. In the MCQ, participants choose between two hypothetical monetary reward options [43]: a smaller reward now (eg, $52 now) or a larger one later (eg, $80 in 50 days). Traditional DDT paradigms lack an objective benchmark for decision-making and are typically monotonous and unengaging for participants. Therefore, gamification has been shown to enhance participant engagement and personal relevance of the DDT, capturing more ecologically valid decision-making and motivation processes [48].

An experiential analogue of the self-report DDT has previously been validated (ie, where an individual must actually experience the time delay), where individuals are required to make decisions on a smaller but closer reward or a larger farther reward in real-time. This paradigm has been shown to be sensitive to substance use disorders such as heroin addiction [48]. The gamified DDT is adapted from this paradigm and involves a coin-hunting game where players are given a set amount of time to earn as many coins as possible. Over 60 trials, players choose between a smaller sum of coins that is closer to their avatar on a grid and, as such, will be faster to reach, or a larger sum of coins that is farther away and takes more time to obtain. The gamified, experiential variant of the DDT takes approximately six minutes to complete. A (*k*) discounting function is computed according to the formula below, along with total coins earned and tally of smaller sooner choices:

V=A(1)1+kD

The (subjective) value of the immediate reward is *V*, value of the delayed reward is *A*, and *D* represents the time delay in days. The outcome variable (*k*), termed the discounting rate, or alternatively described as the indifference point where both rewards become equal in subjective value. Higher *k*-values indicate steeper delay discounting; that is, the subjective value of a reward decreases rapidly across a shorter time span.

### Value-Modulated Attentional Capture Task

The VMAC task measures the tendency to develop reward-related attentional biases [49], akin to sign-tracking in animals [50]. This is a form of conditioned responding thought to reflect a predisposition toward developing addictive behaviours [51, 52]. As such, it was chosen to measure the RDoC constructs of reward learning and compulsivity-related cognitive flexibility. In this task, participants search for a diamond target among circles on each trial. The faster they find and respond to this target, the more points they earn. Critically, one of the (non-target) circles is coloured, either blue or orange (all other shapes are grey) and the colour of this circle—referred to as the distractor—influences the size of the reward available on the current trial, such that one colour (the high-reward colour) signals that a large reward is available, and the other (low-reward) colour signals that a small reward is available. Notably, while the distractor signals reward magnitude, it is never the target that participants respond to in order to receive the reward. Thus, distractors have a Pavlovian, but not instrumental, relation to reward. In ‘sign-trackers’, responses to the target are significantly slower for trials with a high-reward distractor compared to low-reward distractor (ie, the VMAC effect, as indexed by the VMAC score). This suggests that the signal of high reward is more likely to capture participants’ attention, slowing their response to the target – even though this enhanced capture is counterproductive. The VMAC task was recently modified [53] to reflect reward processes specifically [as opposed to the original version, 49, which had a loss component] and extended to include a reversal phase, where the relation between stimulus and reward in the training phase is reversed in the subsequent (reversal) phase. This extension is designed to gauge rigidity and persistence of reward-related attentional biases [53, 54].

The gamified VMAC follows a soccer game format, where players are required to locate a target stimulus, namely a player wearing the same team jersey, while ignoring distractors (players wearing the opposite team jersey). On identifying their teammate, players must pass the ball left or right depending on their teammate’s relative position. On 20 of the 24 trials, the distractors are equally split between two colours, a non-teammate with pink or green hair. These different hair colours signal the magnitude of reward that may be won on that trial. One colour signals high reward (pink), while the other signals low reward (green). For example, if player successfully passes ball to teammate within a second, they will receive 100 points if a pink-haired opponent was on the field and 10 points if a green-haired opponent was on the field. If a ball pass is too slow (ie, 1000 to 2000ms) or wrong, no reward is given. The number of points earned is given in proportion to reaction time, where a shorter reaction time is awarded proportionally with more points. In the reversal phase, these colour-reward contingencies are reversed. There were 6 blocks of trials in total (4 training and 2 reversal). The gamified VMAC-R with six blocks (two reversal) takes approximately 12 minutes to complete. VMAC yields various metrics, including the VMAC training score averaged over training blocks, the VMAC reversal score averaged over reversal blocks, and the total point. A higher VMAC score reflects slower responding to the target in the presence of the high value versus low value distractor. A higher VMAC reversal score reflects slower responding to the previously high value distractor versus the previously low value distractor

### Sequential Decision-Making Task

SDT is a sequential two-step Markov choice task that measures the tendency to rely on model-based (goal-directed) versus model-free (non-goal-directed, habitual) learning [55, 56]. Accordingly, SDT was chosen to measure the constructs of reward learning and habit on the RDoC matrix. SDT has been shown to converge on related individual differences and disorders; for example, higher scores on scales assessing eating disorders, impulsivity, OCD and alcohol misuse were associated with deficits in model-based control on the SDT [57]. Please see supplementary materials for further information on the SDT.

In the gamified version of the SDT, players are presented with an animal rescue task. Their role is to rescue as many animals as they can, who have escaped the animal sanctuary due to a thunderstorm and are hiding in a nearby forest and on a farm. To assist the player in finding the animals, there are four park rangers who will look for the animals in the two different environments. The rangers are divided into two teams, with two rangers on each team. The rangers are easily distinguishable from each other due to differences in clothes, gender and hair colours. On each trial, participants are presented with a team of park rangers and decide which ranger they want to work with on this trial. On each team there is one ranger who will always travel to the forest environment, and one that will always travel to the farmlands, meaning that the transition probability is deterministic, as in the Kool paradigm. Animals appear behind one of two objects in the environment according to the Kool Gaussian random walk. A model-based participant will learn which environment each ranger travels to. When an advantageous outcome is noted in a particular environment (for example, the forest) the model-based participant will become more likely to choose the forest rangers on either team on the next trial. A model-free agent will simply continue to choose the individual ranger they have had previous success with and not conceptualise that the two forest rangers are equally advantageous in this scenario. Players are tasked with trying to rescue as many animals as they can over 125 trials. There is initially a 25-trial practice block. Participants are reminded of the total number of animals rescued throughout the task at regular intervals in the form of a conga line of all animals rescued. The gamified SDT, adapting the Kool version of the paradigm, takes approximately 25 minutes to complete.

SDT yields various metrics. Mixing weight (*w*) shows how model based or free participants were in their decision-making strategy. If *w* = 0, then the participant is completely model free; if *w* = 1, participant is completely model based. Inverse temperature (*β*) is a measure of exploitation, with high levels of *β* denoting more exploitation (more likely to stay), whereas low levels of *β* denoting more exploration (more likely to switch). The learning rate (*α*) is a measure of how much reward prediction error will influence the value assigned to the rangers. If *α* = 1 then *any* positive RPE will make the participant assign all their value to that ranger. If *α* = 0 then RPE will not have any impact on choice. Total points earned was also computed.

### Short UPPS-P Impulsivity Scale [SUPPS-P; 62]

This 20-item scale is a gold standard impulsivity questionnaire with five subscales: Negative Urgency, the tendency toward impulsive action when experiencing strong negative emotions (e.g., ‘When I am upset, I often act without thinking’); Positive Urgency, the tendency toward impulsive action when experiencing strong positive emotions; Lack of Perseverance; Lack of Premeditation; and Sensation Seeking. The present study used total SUPPS-P score.

### The Cambridge-Chicago Compulsivity Trait Scale [CHI-T; 63, 64]

This 15-item scale covers broad aspects of compulsivity, including the need for completion or perfection, being stuck in a habit, reward-seeking, desire for high standards, and avoidance of situations that are hard to control. A total score was computed.

### Impulsive-Compulsive Behaviours Checklist [ICBC; 65]

This scale individually quantifies 33 impulsive and compulsive symptoms on a scale of never, sometimes, often or always; for example, it includes impulse control problems (gambling, substance use, aggression, etc.) and compulsive problems (eg, washing, checking, making lists, counting, etc.). A separate subscore is calculated for impulsions and compulsions.

### Alcohol Use Disorders Identification Test [AUDIT; 66]

The AUDIT is a 10-item measure assessing risky alcohol consumption. The total AUDIT score as computed, with a higher score indicating more hazardous alcohol consumption.

### Kessler-10 item psychological distress scale [K10; 67]

This is a 10-item scale designed to measure psychological distress. A total K10 score was computed, with a higher score indicating greater psychological distress.

### Statistical Analysis

Data were analysed in SPSS V27.0. All cognitive variables were cleaned according to effort and validation checks typical in mechanical turk studies [ie, attention and validation questions; 68]. There were four validity questions (eg, “please ignore the question below and do not answer it”, whereby participants who were responding with minimal effort would have likely selected a response). We also excluded neurocognitive data based on consensus recommendations for the original paradigms (SST and VMAC had standard checking procedures applied). Specifically, SST scores were excluded if participants performed worse than 90% on proportion correct in go trials, or if their correct inhibition performance on stop trials was less than 25% or greater than 75% (indicating ineffective stair-casing). In VMAC, data were excluded if participants performed below chance on the training or reversal blocks. Within-subject analyses of variance were conducted to examine mean differences in key output metrics between the gamified and standard (ie, non-gamified) paradigms. Bivariate correlations were further conducted to determine the magnitude of concordance between the gamified and standard paradigms, with Pearson’s coefficients for normally distributed data and Kendall’s tau for non-normality. Bivariate correlations were also conducted to determine associations between the paradigms and trait-based as well as real-world behavioural measures. Intraclass correlation coefficient (ICC) was used to determine test-retest reliability using an absolute agreement, mixed-effects random model. Bivariate correlations of .1 were interpreted as a small effect; 0.3 as a medium effect; 0.5 as a large effect. ICC values of 0.5 to 0.75 were considered moderate reliability, 0.75 to 0.90 were considered good reliability, and above 0.90 were considered excellent reliability.

## Results

### Sample Characteristics

In total, 556 individuals were included in the final validation sample after data cleaning (94 participants excluded). Of these 197 completed the BART and SST study, 175 completed the DDT and VMAC study, and 184 completed the SDT study. Mean age was 34.8 years old (SD=8.4); 255 were female (45.8%), 5 were “other”; and the sample represented a diverse spread of ethnicities (Caucasian = 399, Hispanic = 37, African American = 62, Asian = 51, Native American = 5, Other = 2). Mean age, as well as sex and ethnicity distribution did not differ among the three validation samples (*P* >.05). Approximately 25% of the overall cohort reported a lifetime history of a mental health or substance use problem (N=140). Whereas both lifetime diagnosis and K-10 score (mean = 18.4) did not differ among samples, the DDT/VMAC and SDT samples reported fewer number of current mental health or substance use diagnoses [16.43% vs. 20.81%; χ^2^(1,2) = 6.78, *P* = .03; 4.9 vs 3.5], but slightly higher AUDIT scores [F(2,553) = 4.35, *P* =.01] than the BART/SST sample. The DDT/VMAC and SDT samples also had greater educational attainment, with 62.85% having a bachelor’s degree compared with 50.25% in the BART/SST sample [χ^2^(1,2) = 9.29, *P* = .01]. For the test-retest sample (N=43), mean age (31.3 years, SD = 11.7) and gender distribution (44% female, n=19) did not significantly differ with the validation samples and therefore are demographically comparable.

### Balloon Analogue Risk Task

#### Internal metrics

**Fig. 2:**
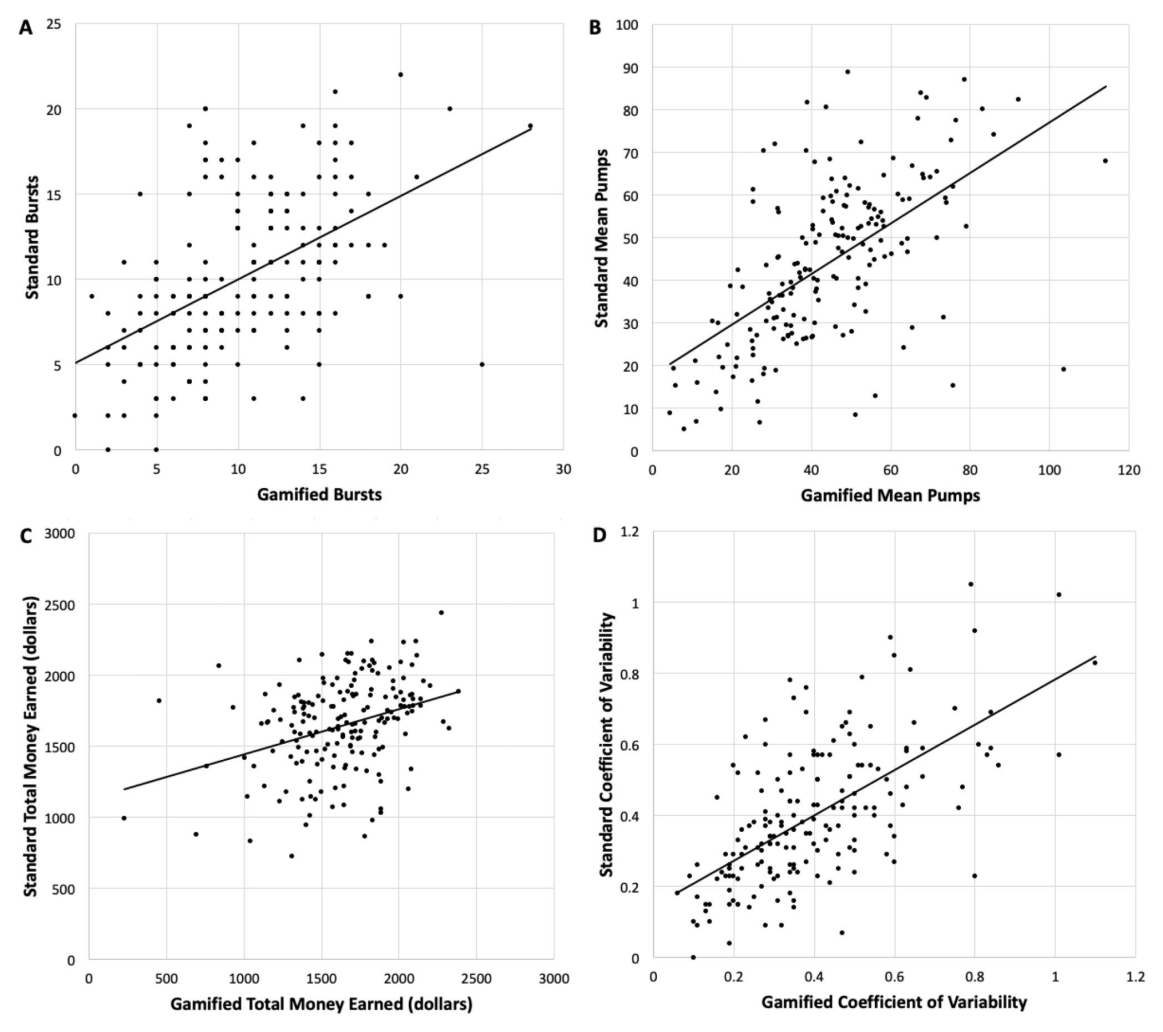
Bivariate correlations between the gamified and standard BART paradigms on bursts (*A*), mean pumps (*B*), total money earned (*C*), and coefficient of variability (*D*)

Bursts, mean pumps, total money earned, and the coefficient of variability all did not significantly differ between the gamified and standard BART (p’s > 0.05; see Table 1). The gamified BART was significantly associated with all corresponding metrics of the standard BART (Fig. 2).

- *Bursts*, r = 0.50, *P* < .001
- *Mean pumps*, r = 0.58, *P* < .001
- *Total money earned*, r = 0.34, *P* < .001
- *Coefficient of variability*, r = 0.63, *P* < .001

#### Trait associations

The coefficient of variability in the gamified BART was significantly and negatively associated with CHI-T score (r = -0.16, *P* = .03). No other gamified or non-gamified BART metric was associated with trait-based metrics.

#### Behavioral correlates

On the gamified BART, bursts, mean pumps and total money earned were associated with ICBC compulsive behaviours (r = 0.18, *P* = 0.01; r = 0.23, p = 0.002; r = -0.17, *P* = .03). Mean pumps on the gamified BART were also associated with ICBC impulsive behaviours (r = 0.147, *P* = 0.048). Bursts on the standard BART were associated with the total AUDIT (r = -0.15, *P* = 0.04). No other metrics were associated with behavioural outcomes on either BART versions.

### Stop Signal Task

#### Internal metrics

**Fig. 3:**
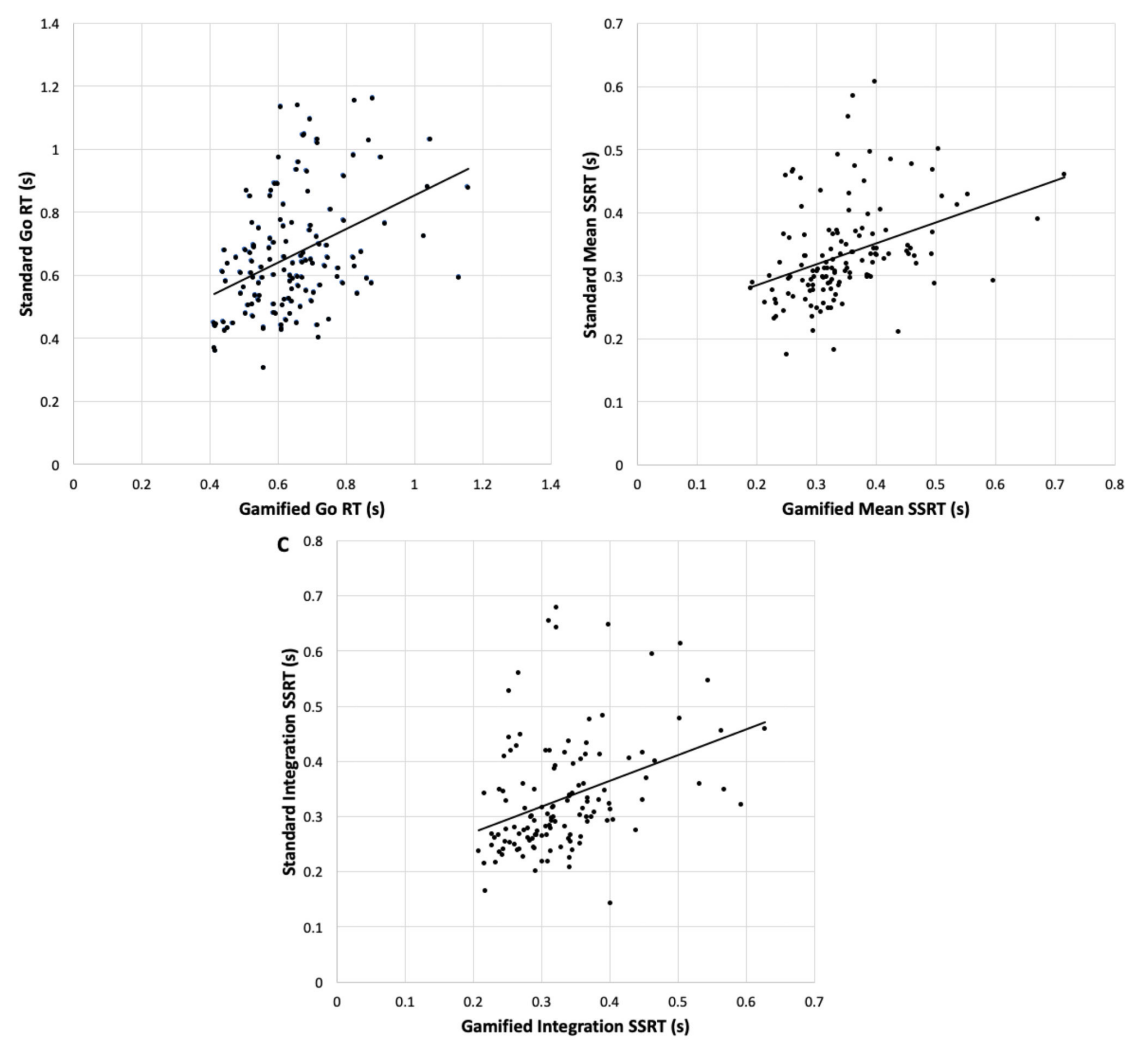
Bivariate correlations between the gamified and standard SST paradigms on Go RT (*A*), Mean SSRT (*B*), and Integration SSRT (*C*)

Go RT, mean SSRT and integration SSRT all did not differ between gamified and standard SST (*P* > .05). All three indices were significantly associated with one another (see Fig. 3).

- *Go RT*, r = 0.40, *P* < .001
- *Mean SSRT*, r = 0.37, *P* < .001
- *Integration SSRT*, r = 0.37, *P* = .001

#### Trait associations

No SSRT score on the gamified or standard SST were associated with trait-based scales (*P* > .05).

#### Behavioral correlates

Mean SSRT was significantly and positive associated with ICBC impulsive behaviours on the gamified SST (r = 0.17, *P* < .05). No other SSRT score on the gamified or standard SST were associated with other behavioural measures.

### Delay Discounting Task

#### Internal metrics

The *k* function was significantly steeper in the gamified DDT than the standard DDT [F(1,174) = 39.47, *P* < .001]. On average, individuals selected the smaller sooner reward more often on the standard MCQ (59.64%) than in the gamified DDT (44.04%; F(1,174) = 33.22, *P* < .001). The k function in the gamified DDT was not significantly associated with the k function on the MCQ. However, total coins earned and proportion of smaller sooner responses on the gamified DDT was associated with the *k* function on the MCQ (r = -0.16, *P* = 0.04; r = 0.15, *P* = 0.05). No other gamified and standard metrics were significantly associated with one another (*P* > 0.05).

#### Trait associations

The MCQ *k* function and proportion of smaller soon responses on the gamified DDT were both associated with SUPPS-P lack of perseveration (r = -0.16, *P* = 0.04; r = -0.22, *P* = .004). No other trait associations were significant (*P* > .05).

#### Behavioral correlates

The *k* function in the gamified DDT was associated with the AUDIT score (tau = 0.12, *P* = .03). By comparison, the MCQ *k* function was associated with ICBC compulsive behaviours (r = 0.19, *P* = .02), and no other behavioural measures were significantly associated with any other DDT or MCQ metrics.

### Value-Modulated Attentional Capture Task

#### Internal metrics

**Fig. 4:**
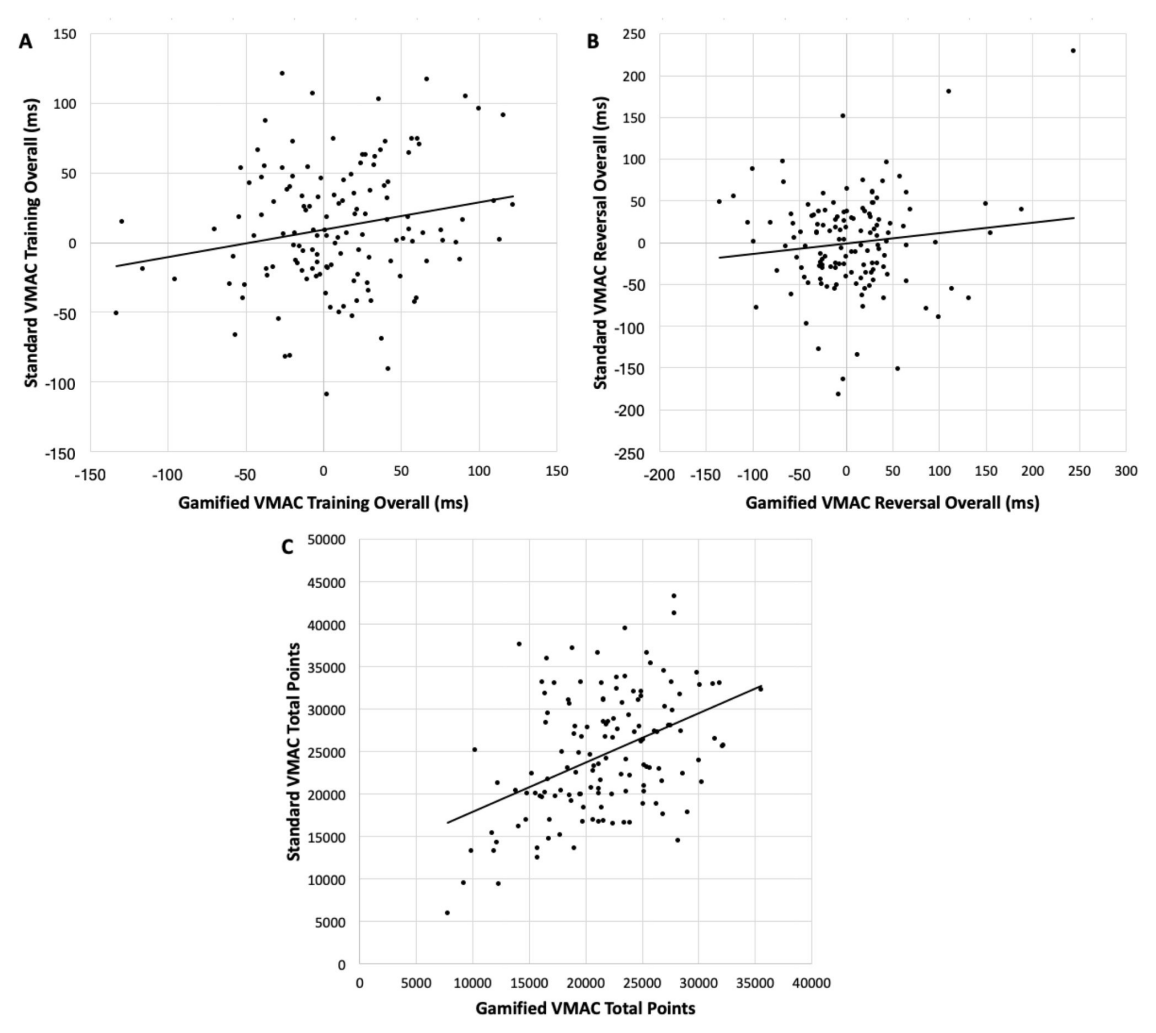
Bivariate correlations between the gamified and standard VMAC paradigms on training overall (*A*), reversal overall (*B*), and total points (*C*)

VMAC total points were significantly higher in the standard VMAC than in the gamified VMAC; F(1, 153) = 26.98, *P* < 0.001). VMAC training score and VMAC reversal score did not differ between gamified and standard variants (*P* > .05). All VMAC indices were either significantly associated with one another or approaching significance (see Fig. 4).

- *VMAC training overall*, r = 0.18, *P* = .04
- *VMAC reversal overall*, r = 0.12, *P* = 0.165
- *VMAC total points*, r = 0.44, *P* < .001

#### Trait associations

VMAC training score on the gamified task was associated with positive and negative urgency subscales on the SUPPS-P (r = -0.16, *P* = .045). All other associations were non-significant.

#### Behavioral correlates

VMAC reversal score on the gamified VMAC was significantly and negatively associated with ICBC compulsive behaviours (r = -0.20, *P* = .01), even after controlling for learning in the final training block in a bootstrapped linear regression (B = 32.90, *P* = .04). No other VMAC scores on either the gamified or standard paradigms were associated with other behavioural scales.

### Sequential Decision-Making Task

#### Internal metrics

Consistent with Kool et al. [59–61], mixing weight (*w*) – or greater model-based decision-making – was significantly associated with greater total score on the gamified SDT (r = 0.32, *P* < .001). This validates the Kool versions’ original assumptions that modifying the task would lead to model-based decision-making becoming more adaptive/advantageous to decision-making (cf. Daw version).

#### Trait associations

(*w*) on the gamified SDT was associated with negative urgency (r = 0.16, *P* = .03). No other trait associations were significant.

#### Behavioral correlates

Most SDT metrics were associated with ICBC compulsive and impulsive behaviours as follows:

- *SDT inverse temperature and ICBC compulsions*, r = 0.29, *P* < .001
- *SDT inverse temperature and ICBC impulsions*, r = 0.28, *P* < .001
- *SDT learning rate and ICBC compulsions*, r = -0.30, *P* < .001
- *SDT learning rate and ICBC impulsions*, r = -0.27, *P* < .001
- *SDT weight parameter and ICBC compulsions*, r = -0.30, *P* < .001
- *SDT weight parameter and ICBC impulsions*, r = -0.26, *P* < .001
- *SDT total points and ICBC compulsions*, r = -0.21, *P* = .004
- *SDT total points and ICBC compulsions*, r = -0.23, *P* = .002

No other gamified SDT metrics were associated with other behavioral scales.

### Test-Retest Reliability

The 3-day test-retest for the gamified tasks in a separate mechanical turk sample (N = 43) were adequate for SST (integration SSRT; ICC = .72, *P* < .001), good for DDT (*k* function; ICC = 0.77, *P* < .001) and SDT (learning rate; ICC = 0.86, *P* < .001), and excellent for VMAC (total points; ICC = 0.91, *P* < .001). By contrast, test-retest reliability for BART (mean pumps) and the remaining VMAC and SDT indices were poor and non-significant (*P* > .05).

## Discussion

### Principal Findings

Two overall findings underscore the validity of the gamified BrainPAC tasks. Firstly, most psychometric outputs (except DDT k function and VMAC total points) did not differ between the gamified and original paradigms. Secondly, the small-to-large associations amongst key output metrics between these versions suggested that the gamified BART, SST, VMAC and SDT appear to be convergent with established measures, albeit smaller in effect than hypothesized. In keeping with the existing literature, the gamified versions of the learning paradigms VMAC and SDT were weakly associated with impulsive traits or not at all (BART, SST, DDT), underscoring the need to measure neurocognition in addition to trait risks. Although the gamified DDT was not significantly associated with the MCQ questionnaire, similar to the gamified BART it was sensitive to alcohol use problems. Further, the experiential version of our gamified temporal discounting task was significantly distinct from the established, non-gamified hypothetical delay discounting, with each being associated with unique outcomes and suggests they are measuring distinct constructs, or aspects thereof. Interestingly, however, it appears that the gamified paradigms have stronger correlations with real-world compulsive and impulsive behaviours than the non-gamified paradigms. Specifically, BART and VMAC were significantly associated with compulsive behaviors, whereas BART and SST were associated with impulsive behaviors, and may reflect enhanced sensitivity to real world actions.

In addition, some key metrics from the BART, SST and DDT demonstrated adequate to excellent test-retest reliability and suggest that they are not only reliable measures but appropriate for risk monitoring over time. Although it stands to be replicated, the lack of test-retest for the VMAC and VMAC-R scores may be an artefact of the compounding of error variability as a result of computing difference scores [69]. Separately, the lack of test-retest for the SDT is not surprising given that – of the five paradigms chosen - SDT is the only task where this has not been established in the original non-gamified paradigm and warrants further study.

### Comparison to Prior Work and Implications

Very few prior studies have examined the validity and reliability of a gamified approach to cognitive assessment with the purpose of developing a comprehensive battery targeting a broad range of addictive behaviors. To our knowledge, this is the first time a comprehensive, gamified battery has been developed for the neurocognitive assessment of addictive behaviors [see, for example, a gamified battery focusing on impulsivity; 70]. The validity and reliability of the gamified tasks ‘is comparable to those in prior studies that have adapted similar cognitive neuroscience paradigms [71–76]. However, whereas past gamification studies primarily looked at demographic associations, the current study investigated convergent validity, potential predictive validity with clinical and behavioural outcomes, as well as test-retest reliability. To a large extent, our gamificated tasks appear to suggest that the BrainPAC measures are sufficiently valid for use in cognitive assessment, even in the context of online testing, and suggests that techniques that putatively enhance engagement may have the potential to yield meaningful assessment outcomes in unsupervised testing. Whether this is due to enhanced engagement warrants specific testing that explicitly measures motivation, as well as by comparison of supervised versus unsupervised test conditions.

The current findings have significant implications for how the addiction field can articulate the current armamentarium of cognitive tools. Most tasks gamified here were, in their original forms, either well-validated by decades of research (ie, SST, BART, DDT) or amassed a consistent empirical consensus from various research groups (ie, VMAC, SDT). Despite the existence of these individual laboratory tools, a comprehensive battery of these tasks has not been developed, validated and made available. Moreover, there are currently no readily available versions of these tasks that are developed for use unsupervised and in a gamified format.

Current findings also suggest that our gamified DDT needs revision. The steeper discounting observed on the MCQ compared to the gamified DDT indicates that the experiential aspect of our paradigm did not achieve the same magnitude of delay as in the hypothetical paradigm. That is, people were more likely to discount rewards in the questionnaire than the gamified task because the ‘cost’ of time was greater. That is, we were unable to sufficiently mimic the disincentivising nature of ‘waiting for larger rewards’ in the short timespan afforded to us by a brief neurocognitive task. Ongoing task development efforts will benefit from examining the effects of administering gamified tasks for brief periods over a number of days and embedding an experiential discounting paradigm across a longer, and perhaps more ecologically valid, timespan.

As expected, the less a paradigm is gamified (eg, BART), the greater the magnitude of association between the gamified and original variants. Whereas, when the gamified task was a significant departure from the original paradigm – as was the case for the gamified DDT, which was an experiential task rather than a hypothetical task as in the MCQ questionnaire – the two tasks were not significantly associated. Despite this reduction in correlation, the gamified DDT remained sensitive to behavioral outcomes (ie, alcohol use), suggesting that experiential discounting tasks is likely to have unique predictive power relevant, real-world behavior. Nevertheless, the correspondence between the gamified and non-gamified tasks were a little less than would be expected and, as such, the further development of the BrainPAC tasks will need to focus on improving this association. It should be acknowledged, however, that a close-to-perfect correlation is not expected given the additional gamified elements that, as a necessity, introduce other cognitive demands.

### Limitations and Future Directions

First, given considerations regarding length of assessment and fatigue, we did not include an in-depth suite of behavioural measures (eg, other substance use), which would have permitted a more comprehensive external validation of the BrainPAC tasks. Second, we were also unable to include all gamified tasks within a single sample due to constraints around having to already administer both versions of every task to the same participants. Third, the identified associations between measures may have been inflated due to shared measurement variance between the gamified and non-gamified tasks (rather than substantive construct-related variance), as well as type I error rates (due to the number of comparisons). As such, these findings stand to be replicated. Fourth, sample characteristics differed slightly according to the time point of data collection, as may be expected (for example due to the COVID pandemic). Future studies should also consider recruiting larger, more population-representative samples for use as normative comparisons.

In the development of the BrainPAC gamified tasks, we had consulted with a cohort of users in the general community in the form of focus groups and qualitative interviews to assist us in developing tasks that individuals would find engaging. However, for the process to be maximally informed by diverse stakeholders, future refinement and iteration of the BrainPAC tasks would require further, more extensive and continual consultation with key users and stakeholders (eg, clinical groups, community cohorts, clinicians) to further refine the BrainPAC tool as an engaging mHealth tool that users would actively engage with in the testing and monitoring of addiction risk and relapse, as well as neurocognitive functions that impact on those outcomes.

## Supplementary Material

Appendix 1

Appendix 2

## Figures and Tables

**Table 1:** Means and standard deviations of key neurocognitive metrics across the gamified and non-gamified tasks

		Gamified		Non-Gamified		Inferential statistics	
		M	SD	M	SD	F statistic	*P*
**BART**	*Bursts*	4.85	10.3	4.62	10.2	0.30	.58
	*Mean pumps*	84.48	8.8	84.23	9	0.14	.71
	*Total money earned*	1641.26	347.9	1645.18	318.6	0.16	.69
	*Coefficient of variability*	0.41	0.2	0.42	0.2	1.44	.23
**SST**	*Go RT*	654.14	145.4	669.54	194	1.43	.23
	*Mean SSRT*	347.52	86.6	338.98	83.1	2.66	.11
	*Integration SSRT*	344.19	106.1	355.11	134.6	0.42	.52
**DDT**	*k function*	0.17	0.3	0.04	0.3	39.47	<.001
**VMAC**	*Training overall*	8.41	48.8	10.32	44.8	0.23	0.64
	*Reversal overall*	5.40	60.8	-1.49	58.1	0.90	0.34
	*Total points*	21006.57	5760.1	24760.59	7192.8	26.98	<.001
**SDT**	*Inverse temperature*	1.96	1.4	-	-	-	-
	*Learning rate*	0.36	0.4	-	-	-	-
	*Weight parameter*	0.47	0.4	-	-	-	-
	*Total points*	591.1	82	-	-	-	-

## Summary

On balance, the digital, gamified assessment of neurocognitive risk factors for addiction appears to be feasible and empirically supported approach, which has the potential for use in scalable mHealth tools. Critically, the psychometrics of BrainPAC tasks appear to be comparable to the corresponding non-gamified assessment paradigms. It stands to be demonstrated how more sophisticated modelling in larger cohorts with more comprehensive measures of behavior can delineate how we can best measure the underlying constructs, further optimise the gamified tasks, and provide useful assessment and monitoring of brain and mental health outcomes in clinical and other relevant settings.

## Abbreviations

AUDIT: alcohol use disorder identification test
BART: balloon analogue risk task
BrainPAC: BrainPark assessment of cognition
CHI-T: the Cambridge-Chicago compulsivity trait scale
DDT: delay discounting task
ICBC: impulsive-compulsive behaviour checklist
ICC: intraclass correlation coefficient
K10: Kessler-10 item psychological distress scale
MCQ: monetary choice questionnaire
OCD: obsessive-compulsive disorder
RDoC: research domain criteria
SDT: sequential decision-making task
SST: stop signal task
SUPPS-P: short UPPS-P impulsivity scale
VMAC: value-modulated attentional capture task
