# A Novel, Expert-Endorsed, Neurocognitive Digital Assessment Tool for Addictive Disorders: Development and Validation Study

**DOI:** 10.2196/44414

**Published:** 2023-08-25

**Authors:** Rico S C Lee, Lucy Albertella, Erynn Christensen, Chao Suo, Rebecca A Segrave, Maja Brydevall, Rebecca Kirkham, Chang Liu, Leonardo F Fontenelle, Samuel R Chamberlain, Kristian Rotaru, Murat Yücel

**Affiliations:** 1 BrainPark Turner Institute for Brain and Mental Health Monash University Clayton Australia; 2 Melbourne School of Psychological Sciences University of Melbourne Parkville Australia; 3 Obsessive, Compulsive, and Anxiety Spectrum Research Program Institute of Psychiatry Federal University of Rio de Janeiro Rio de Janeiro Brazil; 4 D'Or Institute for Research and Education Rio de Janeiro Brazil; 5 Department of Psychiatry University of Southampton Southampton United Kingdom; 6 Monash Business School Monash University Clayton Australia; 7 QIMR Berghofer Medical Research Institute Herston Australia

**Keywords:** cognitive neuroscience, addictive behaviors, mental health, gaming, gamified, gamification, development, assessment, software, addiction, mental disorder, neurocognition, neurocognitive, brain health, gamified task, psychometric, game developer, game development, validation, validate, validity

## Abstract

**Background:**

Many people with harmful addictive behaviors may not meet formal diagnostic thresholds for a disorder. A dimensional approach, by contrast, including clinical and community samples, is potentially key to early detection, prevention, and intervention. Importantly, while neurocognitive dysfunction underpins addictive behaviors, established assessment tools for neurocognitive assessment are lengthy and unengaging, difficult to administer at scale, and not suited to clinical or community needs. The BrainPark Assessment of Cognition (BrainPAC) Project sought to develop and validate an engaging and user-friendly digital assessment tool purpose-built to comprehensively assess the main consensus-driven constructs underpinning addictive behaviors.

**Objective:**

The purpose of this study was to psychometrically validate a gamified battery of consensus-based neurocognitive tasks against standard laboratory paradigms, ascertain test-retest reliability, and determine their sensitivity to addictive behaviors (eg, alcohol use) and other risk factors (eg, trait impulsivity).

**Methods:**

Gold standard laboratory paradigms were selected to measure key neurocognitive constructs (Balloon Analogue Risk Task [BART], Stop Signal Task [SST], Delay Discounting Task [DDT], Value-Modulated Attentional Capture [VMAC] Task, and Sequential Decision-Making Task [SDT]), as endorsed by an international panel of addiction experts; namely, response selection and inhibition, reward valuation, action selection, reward learning, expectancy and reward prediction error, habit, and compulsivity. Working with game developers, BrainPAC tasks were developed and validated in 3 successive cohorts (total N=600) and a separate test-retest cohort (N=50) via Mechanical Turk using a cross-sectional design.

**Results:**

BrainPAC tasks were significantly correlated with the original laboratory paradigms on most metrics (*r*=0.18-0.63, *P*<.05). With the exception of the DDT k function and VMAC total points, all other task metrics across the 5 tasks did not differ between the gamified and nongamified versions (*P*>.05). Out of 5 tasks, 4 demonstrated adequate to excellent test-retest reliability (intraclass correlation coefficient 0.72-0.91, *P*<.001; except SDT). Gamified metrics were significantly associated with addictive behaviors on behavioral inventories, though largely independent of trait-based scales known to predict addiction risk.

**Conclusions:**

A purpose-built battery of digitally gamified tasks is sufficiently valid for the scalable assessment of key neurocognitive processes underpinning addictive behaviors. This validation provides evidence that a novel approach, purported to enhance task engagement, in the assessment of addiction-related neurocognition is feasible and empirically defensible. These findings have significant implications for risk detection and the successful deployment of next-generation assessment tools for substance use or misuse and other mental disorders characterized by neurocognitive anomalies related to motivation and self-regulation. Future development and validation of the BrainPAC tool should consider further enhancing convergence with established measures as well as collecting population-representative data to use clinically as normative comparisons.

## Introduction

Addiction is defined as a chronic relapsing disorder characterized by compulsive substance seeking or other compulsive behaviors (eg, problem gambling) and is associated with an average loss of 14 years in life expectancy [1]. A key mechanism impacting addiction risk is cost-benefit decision-making ability [2]. The neurocognitive processes underpinning choice behaviors are complex and dynamic with distinct changes corresponding to differing stages of the addiction cycle (eg, transition from impulsivity to compulsivity) [3]. Despite our decades-old understanding of the cognitive mechanisms central to the development and maintenance of addiction, predominantly from preclinical research [4], but increasingly in the clinical neurosciences [5], there is currently no purpose-built tool that comprehensively indexes key neurocognitive functions for people on the addiction spectrum. A comprehensive tool has the potential to facilitate early risk detection and monitoring in addiction as well as identify targets for early intervention and measure the potential cognitive effects of interventions. Additionally, there is an imperative for approaches that are more engaging than laboratory or pen-and-paper clinical tasks (eg, digital, user-friendly, or gamified), given that neurocognitive testing is particularly vulnerable to poor effort, especially when unsupervised. There is also a significant need for scalable, potentially “self-administered” assessment technologies accessible to more people who may have limited access to traditional clinical services [6-9]. As such, an engaging and scalable (ie, mobile health [mHealth]) tool designed to validly and sensitively measure neurocognitive and motivational risk for addictive behaviors is critical to meeting this unmet demand.

Current neurocognitive batteries are limited by their focus on functions that are not core to addiction. These functions were typically selected from neuropsychological tests intended for use in brain injury and neurological disorders, such as processing speed, attention span, episodic memory, and nonverbal reasoning, among others [10,11]. Despite some being associated with risk for certain substance use disorders (eg, working memory and alcohol addiction) [12], they are not broadly thought to be mechanistically and causally linked to the perpetuation of substance and behavioral addictions and, as such, cannot inform risk detection. That is, current approaches focus on problematic addictive behaviors (eg, alcohol misuse) and their neurocognitive sequelae (eg, memory impairment) rather than the underlying motivational or top-down neurocognitive antecedent processes known to drive those behaviors (eg, inhibitory dyscontrol) [9].

By contrast, neurocognitive functions subserved by the neurocircuitries of motivation and self-regulation, which determine the risk of developing maladaptive “approach behaviors” [7], are mechanistically linked to addictive behaviors. Guided by the National Institute of Mental Health–research domain criteria (RDoC), a 3-round international Delphi consensus study of world-leading addiction experts identified key constructs mechanistically implicated in addiction-related outcomes, namely 5 positive valence constructs (reward valuation, expectancy and reward prediction error, action selection, reward learning, and habit), a cognitive control construct (response selection and inhibition), and a non-RDoC expert-initiated construct (compulsivity) [13]. This is corroborated by the existing addiction neuroscience literature, which has established key risk mechanisms linked to these functions. For example, the preference or reward valuation of a smaller, immediate reward over a larger, later reward is a significant risk factor across a range of addictive behaviors [14,15]. Despite our wealth of knowledge, a key barrier remains: there is currently no psychometrically validated, consensus-driven neurocognitive battery to holistically assess these constructs. Narrow, single-construct approaches are insufficient given the significant overlap between relevant risk domains, which a comprehensive tool can help to disentangle regarding mechanisms (ie, what are unique risk factors).

**Figure 1:**
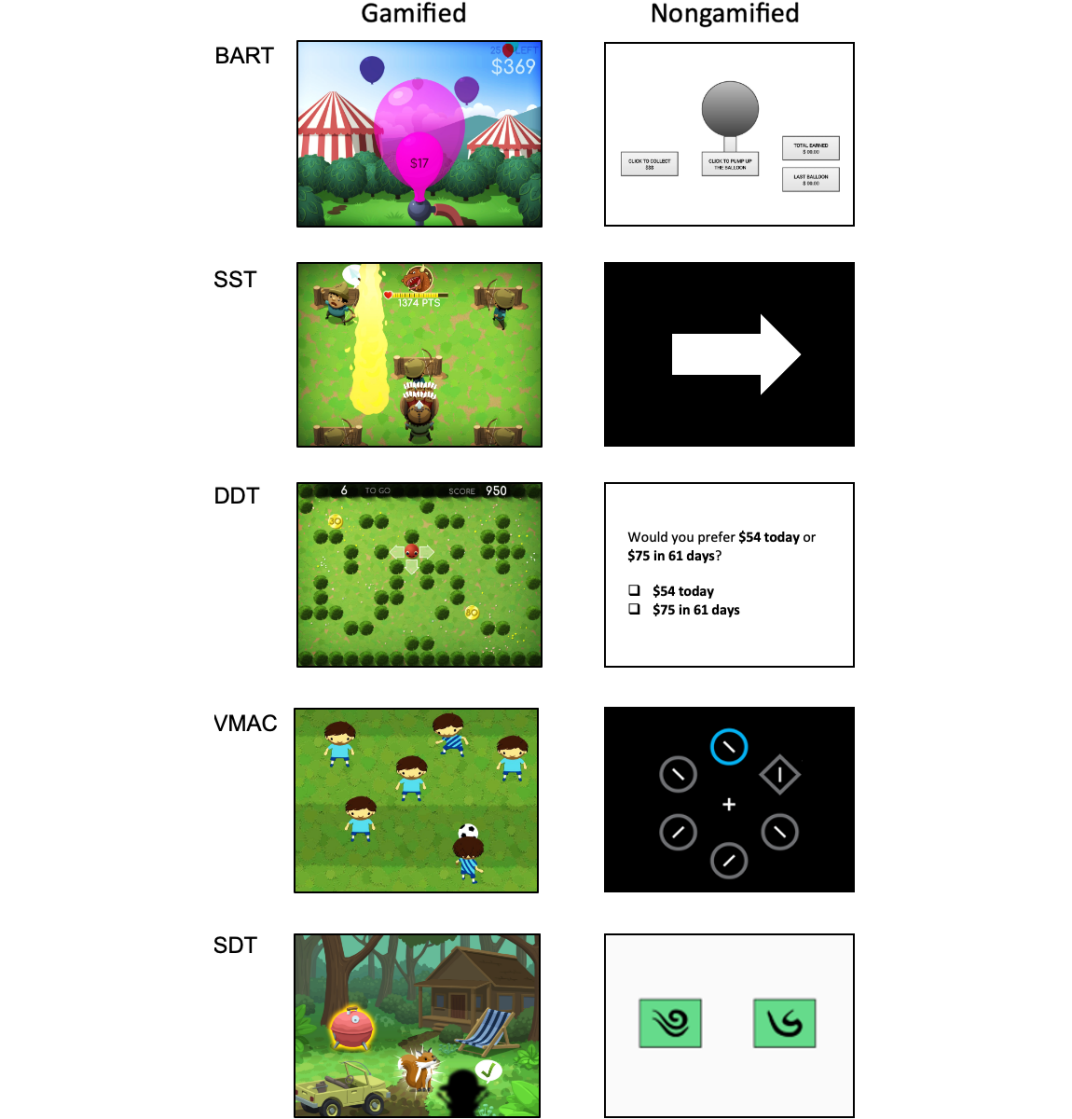
The gamified BrainPark Assessment of Cognition (BrainPAC) tasks compared to the nongamified paradigms. All monetary rewards were in Australian Dollars (approximately Aus $1 was equal to US $0.6477 at the time of the study). BART: Balloon Analogue Risk Task; DDT: Delay Discounting Task; SDT: Sequential Decision-Making Task; SST: Stop Signal Task; VMAC: Value-Modulated Attentional Capture.

To this end, the BrainPark Assessment of Cognition (BrainPAC) digital assessment battery (Figure 1) was developed. Given that accurate neurocognitive assessment relies on people giving strong and enduring effort to the task at hand [16], “gamification” (ie, the redesign of tasks to include game elements) [17] was used with the goal of addressing the potential motivational challenges that stem from unsupervised testing environments and are inherent to app-based assessment tools. Gamification was conducted carefully and iteratively between neuropsychologists (RSCL, RS, and MY), industry partners, and community focus groups over regular workshops to ensure that paradigms continue to retain the requisite neurocognitive elements and, as such, remain valid and reliable while being engaging to end users. Of relevance, there is evidence that gamification can introduce additional cognitive demands [18], and efforts were made at all stages of development to minimize these additional demands. BrainPAC, therefore, represents a shift in how we approach traditional cognitive assessment in that user-friendliness, engagement, and expert endorsement are driving principles in task development. Given the emerging evidence behind the gamified approach, BrainPAC ultimately serves as a proof-of-principle for future mHealth innovations.

Five cognitive paradigms were developed into gamified tasks: the Balloon Analogue Risk Task (BART) to index action selection, the Stop Signal Task (SST) to index response selection and inhibition, Delay Discounting Task (DDT) to index reward valuation, the Value-Modulated Attentional Capture (VMAC) reversal task to index reward learning and compulsivity-related cognitive flexibility, and the Sequential Decision-Making Task (SDT) to index reward learning and expectancy and reward prediction error. In addition to neurocognition, the literature also documents a consistent relationship between trait-based factors (eg, trait impulsivity and trait compulsivity) and addiction risk [3,19-24]. Therefore, the BrainPAC tool goes beyond cognition to include gold standard, structured trait (eg, impulsivity and compulsivity) and behavioral (eg, alcohol use) structured scales [13,25-27] as well as a personalized feedback system that tracks and presents changes over time. However, these latter components are beyond the scope of this current Stage 1 validation, and only the neurocognitive validation data will be presented herein. Stages 2 and 3 of the BrainPAC validation project will address the other components of the BrainPAC tool. In total, the 5 gamified tasks take approximately 60 minutes to complete.

We sought to empirically validate the gamified BrainPAC tasks alongside the original laboratory paradigms, the recommended gold standard approach [17]. We hypothesized that BrainPAC task metrics (ie, subscores from each measure, such as Go Reaction Time on the SST) would not significantly differ from the original laboratory-based task metrics. Further, we predicted that gamified metrics would yield large correlations with the original metrics and would demonstrate expected divergence or small associations with trait-based measures (ie, impulsivity and compulsivity) as well as small to moderate correlations with common addictive behaviors (eg, alcohol use). We chose to focus here on general community samples, irrespective of clinical or diagnostic thresholds.

## Methods

### Recruitment

A total of 650 participants were recruited from Mechanical Turk [28]. Mechanical Turk is a web-based crowdsourcing platform that facilitates rapid recruitment and has been shown to yield more demographically diverse samples than traditional samples and other internet samples [29-31]. Indeed, Mechanical Turk samples tend to include individuals with greater mental health and addiction burdens [29-31]. Importantly, the platform indexes users on the quality of their work by an “approval rating” (ie, percentage of jobs completed satisfactorily), with data showing that restricting recruitment to users with >95% approval ratings yields high-quality data for research [32]. Data quality in this study was further enhanced through the implementation of validity questions, screening for reduced effort, and the exclusion of data from individuals who completed tasks within implausible timeframes. All data collected was nonidentifiable and stored on password-protected local servers, with the investigators having sole access. The inclusion criteria included participants aged 18-55 years who were fluent in English.

### Ethics Approval

This study was approved by the Monash University Human Research Ethics Committee (approval ID: 8239), and all participants gave informed, written consent prior to participation. Participants were reimbursed US $1 for every 10 minutes of research participation.

### Study Design

Participants were recruited in 3 consecutive cohorts for task validation, which coincided with task development milestone dates. The first phase of recruitment occurred in August 2019 (BART and SST); the second (DDT and VMAC) and third (SDT) occurred in May 2020. We chose not to validate all tasks within a single sample to minimize fatigue effects within each cohort since the combined length of tasks was very long as participants completed both the gamified and standard versions of each paradigm. This was done with the exception of the SDT, which is the longest of the 5 tasks, as we already had access to comparable data provided by authors from the nongamified paradigms. The order in which the gamified and standard versions were presented in each cohort was counterbalanced to minimize order effects. A separate sample was recruited to examine test-retest reliability, again with task order counterbalanced.

### Measures

#### BART

The BART indexes action selection within the RDoC matrix. It measures the tendency to make risky decisions and how an individual balances the potential for risk versus reward [33]. Risky decision-making on the BART is linked to real-world risk-taking, including unsafe driving [34], alcohol use [35], drug use [36], and risky sexual behavior [37].

In keeping with the standard paradigm, participants are presented with balloons on the gamified BART and offered the chance to earn money through sequential inflation decisions. Each balloon pump earns the player a potential monetary reward. Once a (predetermined pseudorandomized) burst threshold is reached, the balloon will explode, and all earnings from that balloon are lost. Thus, each pump increases the risk of a loss while at the same time increasing the potential reward. We created a “precommitment” version of the BART, where players have to decide the number of pumps they want to make (ie, risk) immediately upon presentation of each new balloon, rather than over an extended period of individual manual “pumps” (ie, button presses) as per the traditional paradigm [38,39]. This variant has been shown to be a more accurate reflection of risk-taking given the amount of risk a player is willing to take on burst balloons is predetermined, in contrast to the standard, manual “pump BART,” where an intended risk is lost on burst trials [39]. The task was designed so individuals could stretch the balloon to their desired size (potential monetary reward) by keeping a finger pressed on the left mouse key before banking by letting go of the cursor. Three practice trials preceded 30 test trials. The mean burst point was set at 64 pumps (out of 128). The task takes approximately 5 minutes. Four key metrics were computed based on prior studies [39]: (total) bursts, mean (precommitted) pumps, (total) money earned, and coefficient of variability.

#### SST

SST is one of the most commonly used response inhibition tasks [40,41] and was chosen to measure the construct of response selection and inhibition. In this task, participants are asked to press a button (eg, the left button) in response to a stimulus (eg, the left arrow) and another (eg, the right button) in response to another stimulus (eg, the right arrow). This choice reaction time part is known as the “go” trials. On a portion of trials, however, a “stop signal” stimulus (eg, a colored dot or a beep sound) will appear after a delay (known as the “stop signal delay” [SSD]), requiring participants to inhibit an already initiated but latent go-trial “response.” The SSD is stair-cased (ie, difficulty level decreases or increases according to performance) to ensure performance is kept close to 50% correct. A “stop signal reaction time” (SSRT) is computed from the distribution of responses, indexing the average time required to inhibit an initiated go response. Response inhibition on the SST has been associated with a range of addictive behaviors, including alcohol misuse, opioid dependence, and problem gambling [6].

In the gamified SST, players engage in a battlefield game to replenish the arrow supplies of their teammates on the battlefield. Players press left or right to move the character up the grid to restock arrows as quickly as they can when signaled by one of the 2 archers. In a minority of trials (ie, 30%), the enemy dragon breathes fire on the battlefield, necessitating the player to withhold their response (ie, the “stop trials”). We incorporated a reward system to incentivize faster go responses using a points system (and reduce the chance of players “waiting” for the stop signal as a strategy), as previously recommended by a panel of experts [42], whereby greater points were awarded when correct go responses were quicker (up to a maximum of 25 points per trial). A progress bar and sound effects were included to further enhance engagement. There were 10 practice and 150 test trials, with SSD starting at 200 ms and stair-cased by 50 ms. Participants were required to achieve 70% correct go responses before proceeding to test trials (or having to repeat the practice). The gamified SST tasks take approximately 12 minutes. Here we calculated SSRT using both the mean and integration methods [42] as well as computing the mean go reaction time (Go RT).

#### DDT

The monetary choice questionnaire (MCQ) is the most commonly used DDT paradigm [43] and was chosen to index reward valuation, given that temporal discounting is a key and well-understood component of this construct. DDT measures the propensity to seek a reward of lower value that is quicker to obtain over seeking a larger reward that takes more time to acquire [44]. A bias toward instant gratification over long-term goals is characteristic of individuals who are considered impulsive, often contributing to problems such as substance abuse [43], smoking [45], gambling [46], and obesity [47]. In the MCQ, participants choose between 2 hypothetical monetary reward options [43]: a smaller reward now (eg, US $52 now) or a larger one later (eg, US $80 in 50 days). Traditional DDT paradigms lack an objective benchmark for decision-making and are typically monotonous and unengaging for participants. Therefore, gamification has been shown to enhance participant engagement and personal relevance of the DDT, capturing more ecologically valid decision-making and motivation processes [48].

An experiential analog of the self-reported DDT has previously been validated (ie, where an individual must actually experience the time delay), where individuals are required to make decisions on a smaller but closer reward or a larger but farther reward in real time. This paradigm has been shown to be sensitive to substance use disorders such as heroin addiction [48]. The gamified DDT is adapted from this paradigm and involves a coin-hunting game where players are given a set amount of time to earn as many coins as possible. Over 60 trials, players choose between a smaller sum of coins that is closer to their avatar on a grid and, as such, will be faster to reach, or a larger sum of coins that is farther away and takes more time to obtain. The gamified, experiential variant of the DDT takes approximately 6 minutes to complete. A (*k*) discounting function is computed according to the formula below, along with total coins earned and a tally of smaller sooner choices:


V = A (1) 1 + kD

The (subjective) value of the immediate reward is *V*, the value of the delayed reward is *A*, and *D* represents the time delay in days. The outcome variable (*k*) is termed the discounting rate or alternatively described as the indifference point where both rewards become equal in subjective value. Higher *k*-values indicate steeper delay discounting; that is, the subjective value of a reward decreases rapidly across a shorter time span.

#### VMAC Task

The VMAC task measures the tendency to develop reward-related attentional biases [49], akin to sign-tracking in animals [50]. This is a form of conditioned response thought to reflect a predisposition toward developing addictive behaviors [51,52]. As such, it was chosen to measure the RDoC constructs of reward learning and compulsivity-related cognitive flexibility. In this task, participants search for a diamond target among circles on each trial. The faster they find and respond to this target, the more points they earn. Critically, one of the (nontarget) circles is colored, either blue or orange (all other shapes are grey), and the color of this circle—referred to as the distractor—influences the size of the reward available on this trial, such that one color (the high-reward color) signals that a large reward is available and the other (low-reward) color signals that a small reward is available. Notably, while the distractor signals reward magnitude, it is never the target that participants respond to in order to receive the reward. Thus, distractors have a Pavlovian, but not instrumental, relation to reward. In “sign-trackers,” responses to the target are significantly slower for trials with a high-reward distractor compared to trials with a low-reward distractor (ie, the VMAC effect, as indexed by the VMAC score). This suggests that the signal of high reward is more likely to capture participants’ attention, slowing their response to the target, even though this enhanced capture is counterproductive. The VMAC task was recently modified [53] to reflect reward processes specifically (as opposed to the original version [49], which had a loss component) and extended to include a reversal phase, where the relation between stimulus and reward in the training phase is reversed in the subsequent (reversal) phase. This extension is designed to gauge the rigidity and persistence of reward-related attentional biases [53,54].

The gamified VMAC follows a soccer game format where players are required to locate a target stimulus, namely a player wearing the same team jersey, while ignoring distractors (players wearing the opposite team jersey). After identifying their teammate, players must pass the ball left or right depending on their teammate’s relative position. In 20 of the 24 trials, the distractors are equally split between 2 colors: a nonteammate with pink or green hair. These different hair colors signal the magnitude of reward that may be won on that trial. One color signals a high reward (pink), while the other signals a low reward (green). For example, if a player successfully passes the ball to a teammate within a second, they will receive 100 points if a pink-haired opponent is on the field and 10 points if a green-haired opponent is on the field. If a ball pass is too slow (ie, 1000-2000 ms) or wrong, no reward is given. The number of points earned is given in proportion to reaction time, where a shorter reaction time is awarded proportionally with more points. In the reversal phase, these color-reward contingencies are reversed. There were 6 blocks of trials in total (4 training and 2 reversals). The gamified VMAC-R with 6 blocks (2 reversals) takes approximately 12 minutes to complete. VMAC yields various metrics, including the VMAC training score averaged over training blocks, the VMAC reversal score averaged over reversal blocks, and the total point. A higher VMAC score reflects a slower response to the target in the presence of a high-value versus low-value distractor. A higher VMAC reversal score reflects a slower response to the previously high-value distractor versus the previously low-value distractor.

#### SDT

SDT is a sequential 2-step Markov choice task that measures the tendency to rely on model-based (goal-directed) versus model-free (nongoal-directed, habitual) learning [55,56]. Accordingly, SDT was chosen to measure the constructs of reward learning and habit on the RDoC matrix. The SDT has been shown to converge on related individual differences and disorders; for example, higher scores on scales assessing eating disorders, impulsivity, obsessive-compulsive disorder (OCD), and alcohol misuse were associated with deficits in model-based control on the SDT [57]. Please see Multimedia Appendix 1 [55, 58-61] for further information on the Kool version of the SDT, which was adapted in the current study.

In the gamified version of the SDT, players are presented with an animal rescue task. Their role is to rescue as many animals as they can who have escaped the animal sanctuary due to a thunderstorm and are hiding in a nearby forest and on a farm. To assist the player in finding the animals, there are 4 park rangers who will look for them in the 2 different environments. The rangers are divided into 2 teams, with 2 rangers on each team. The rangers are easily distinguishable from each other due to differences in clothes, gender, and hair colors. On each trial, participants are presented with a team of park rangers and decide which ranger they want to work with. On each team, there is one ranger who will always travel to the forest environment and one who will always travel to the farmlands, meaning that the transition probability is deterministic, as in the Kool paradigm. Animals appear behind one of 2 objects in the environment, according to the Kool Gaussian random walk. A model-based participant will learn which environment each ranger travels to. When an advantageous outcome is noted in a particular environment (eg, the forest), the model-based participant will become more likely to choose the forest rangers on either team on the next trial. A model-free agent will simply continue to choose the individual ranger they have had previous success with and not conceptualize that the 2 forest rangers are equally advantageous in this scenario. Players are tasked with trying to rescue as many animals as they can over the course of 125 trials. There is initially a 25-trial practice block. Participants are reminded of the total number of animals rescued throughout the task at regular intervals in the form of a conga line of all animals rescued. The gamified SDT, adapting the Kool version of the paradigm, takes approximately 25 minutes to complete.

SDT yields various metrics. Mixing weight (*w*) shows how model-based or free participants were in their decision-making strategy. If *w*=0, then the participant is completely model-free; if *w*=1, the participant is completely model-based. Inverse temperature (β) is a measure of exploitation, with high levels of β denoting more exploitation (more likely to stay), whereas low levels of β denoting more exploration (more likely to switch). The learning rate (α) is a measure of how much reward prediction error will influence the value assigned to the rangers. If α=1, then any positive RPE will make the participant assign all their value to that ranger. If α=0, then RPE will not have any impact on choice. Total points earned were also computed.

#### Short UPPS-P Impulsivity Scale

Short UPPS-P Impulsivity Scale (SUPPS-P) [62] is a 20-item scale with a gold standard impulsivity questionnaire with five subscales: (1) Negative Urgency, the tendency toward impulsive action when experiencing strong negative emotions (eg, “When I am upset, I often act without thinking”); (2) Positive Urgency, the tendency toward impulsive action when experiencing strong positive emotions; (3) Lack of Perseverance; (4) Lack of Premeditation; and (5) Sensation Seeking. This study used the total SUPPS-P score.

#### The Cambridge-Chicago Compulsivity Trait Scale

The Cambridge-Chicago Compulsivity Trait Scale (CHI-T) [63,64] is a 15-item scale that covers broad aspects of compulsivity, including the need for completion or perfection, being stuck in a habit, reward-seeking, desire for high standards, and avoidance of situations that are hard to control. A total score was computed.

#### Impulsive-Compulsive Behaviors Checklist

This scale [65] individually quantifies 33 impulsive and compulsive symptoms on a scale of never, sometimes, often, or always. For example, it includes impulse control problems (gambling, substance use, aggression, etc) and compulsive problems (eg, washing, checking, making lists, and counting). A separate subscore is calculated for impulsions and compulsions.

#### Alcohol Use Disorders Identification Test

The Alcohol Use Disorders Identification Test (AUDIT) [66] is a 10-item measure assessing risky alcohol consumption. The total AUDIT score was computed, with a higher score indicating more hazardous alcohol consumption.

#### Kessler 10-Item Psychological Distress Scale

This is a 10-item scale [67] designed to measure psychological distress. A total Kessler 10-Item Psychological Distress Scale (K10) score was computed, with a higher score indicating greater psychological distress.

### Statistical Analysis

The data were analyzed in SPSS (version 27.0; IBM Corp). All cognitive variables were cleaned according to effort and validation checks typical in Mechanical Turk studies (ie, attention and validation questions) [68]. There were 4 validity questions (eg, “please ignore the question below and do not answer it,” whereby participants who were responding with minimal effort would have likely selected a response). We also excluded neurocognitive data based on consensus recommendations for the original paradigms (SST and VMAC had standard checking procedures applied). Specifically, SST scores were excluded if participants performed worse than 90% on proportion correct in go trials, or if their correct inhibition performance on stop trials was less than 25% or greater than 75% (indicating ineffective stair-casing). In VMAC, data were excluded if participants performed below chance on the training or reversal blocks. Within-subject analyses of variance were conducted to examine mean differences in key output metrics between the gamified and standard (ie, nongamified) paradigms. Bivariate correlations were further conducted to determine the magnitude of concordance between the gamified and standard paradigms, with Pearson coefficients for normally distributed data and Kendall tau for nonnormality. Bivariate correlations were also conducted to determine associations between the paradigms and trait-based as well as real-world behavioral measures. The intraclass correlation coefficient (ICC) was used to determine test-retest reliability using an absolute agreement, mixed-effects random model. Bivariate correlations of 0.1 were interpreted as a small effect; 0.3 as a medium effect and 0.5 as a large effect. ICC values of 0.5 to 0.75 were considered moderate reliability, 0.75 to 0.90 were considered good reliability, and above 0.90 were considered excellent reliability.

## Results

### Sample Characteristics

In total, 556 individuals were included in the final validation sample after data cleaning (94 participants were excluded). Of these, 197 completed the BART and SST study, 175 completed the DDT and VMAC study, and 184 completed the SDT study. The mean age was 34.8 (SD 8.4) years; 255 (45.8%) were female, 5 were “other,” and the sample represented a diverse spread of ethnicities (Caucasian n=399, Hispanic n=37, African American n=62, Asian n=51, Native American n=5, other n=2). The mean age, as well as sex and ethnicity distributions, did not differ among the 3 validation samples (*P*>.05). Approximately 25% (n=140) of the overall cohort reported a lifetime history of a mental health or substance use problem. Whereas both lifetime diagnosis and K-10 score (mean 18.4) did not differ among samples, the DDT/VMAC and SDT samples reported fewer numbers of current mental health or substance use diagnoses (16.43% vs 20.81%; *χ*^2^_1,2_=6.78, *P*=.03; 4.9 vs 3.5), but slightly higher AUDIT scores (*F*_2,553_=4.35, *P*=.01) than the BART/SST sample. The DDT/VMAC and SDT samples also had greater educational attainment, with 62.85% having a bachelor’s degree compared with 50.25% in the BART/SST sample (*χ*^2^_1,2_=9.29, *P*=.01). For the test-retest sample (n=43), mean age (31.3, SD 11.7 years) and gender distribution (n=19, 44% female) did not significantly differ with the validation samples and therefore are demographically comparable. Please see Multimedia Appendix 2 for additional data on sample characteristics as well as exploratory bivariate correlations.

### BART

#### Internal Metrics

**Figure 2:**
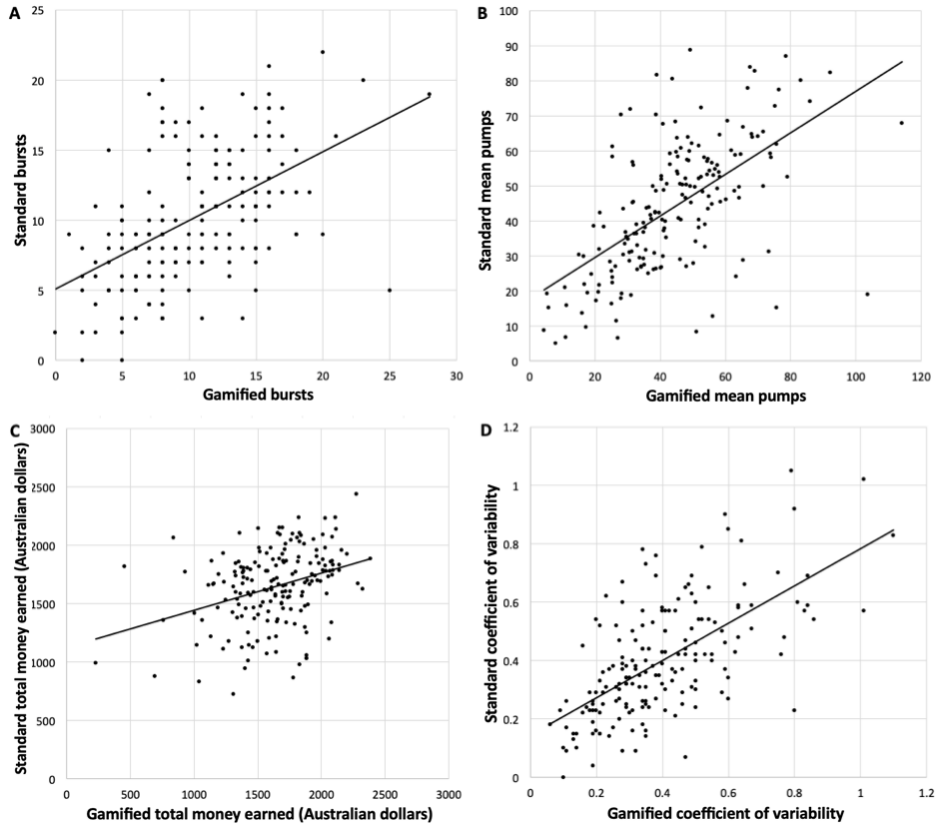
Bivariate correlations between the gamified and standard Balloon Analogue Risk Task (BART) paradigms on (A) bursts, (B) mean pumps, (C) total money earned, and (D) coefficient of variability.

Bursts, mean pumps, total money earned, and the coefficient of variability did not significantly differ between the gamified and standard BART (*P*>.05; see Table 1). The gamified BART was significantly associated with all corresponding metrics of the standard BART (Figure 2).

- Bursts: *r*=0.50, *P*<.001
- Mean pumps: *r*=0.58, *P*<.001
- Total money earned: *r*=0.34, *P*<.001
- Coefficient of variability: *r*=0.63, *P*<.001

**Table 1:** Means and SDs of key neurocognitive metrics across the gamified and nongamified tasks.

		Gamified, mean (SD)	Nongamified, mean (SD)	Inferential statistics	
				*F* statistic	*P* values
**BART^a^**					
	Bursts	4.85 (10.3)	4.62 (10.2)	0.30	.58
	Mean pumps	84.48 (8.8)	84.23 (9)	0.14	.71
	Total money earned	1641.26 (347.9)	1645.18 (318.6)	0.16	.69
	Coefficient of variability	0.41 (0.2)	0.42 (0.2)	1.44	.23
**SST^b^**					
	Go RT^c^	654.14 (145.4)	669.54 (194)	1.43	.23
	Mean SSRT^d^	347.52 (86.6)	338.98 (83.1)	2.66	.11
	Integration SSRT	344.19 (106.1)	355.11 (134.6)	0.42	.52
**DDT^e^**					
	k function	0.17 (0.3)	0.04 (0.3)	39.47	<.001
**VMAC^f^**					
	Training overall	8.41 (48.8)	10.32 (44.8)	0.23	.64
	Reversal overall	5.40 (60.8)	–1.49 (58.1)	0.90	.34
	Total points	21,006.57 (5760.1)	24,760.59 (7192.8)	26.98	<.001
**SDT^g^**					
	Inverse temperature	1.96 (1.4)	N/A^h^	N/A	N/A
	Learning rate	0.36 (0.4)	N/A	N/A	N/A
	Weight parameter	0.47 (0.4)	N/A	N/A	N/A
	Total points	591.1 (82)	N/A	N/A	N/A

^a^BART: Balloon Analogue Risk Task.

^b^SST: Stop Signal Task.

^c^Go RT: go reaction time.

^d^SSRT: stop signal reaction time.

^e^DDT: Delay Discounting Task.

^f^VMAC: Value-Modulated Attentional Capture.

^g^SDT: Sequential Decision-Making Task.

^h^N/A: not applicable.

#### Trait Associations

The coefficient of variability in the gamified BART was significantly and negatively associated with the CHI-T score (*r*=–0.16, *P*=.03). No other gamified or nongamified BART metric was associated with trait-based metrics.

#### Behavioral Correlates

On the gamified BART, bursts, mean pumps, and total money earned were associated with ICBC compulsive behaviors (*r*=0.18, *P*=.01; *r*=0.23, *P*=.002; *r*=–0.17, *P*=.03). Mean pumps on the gamified BART were also associated with ICBC impulsive behaviors (*r*=0.147, *P*=.048). Bursts on the standard BART were associated with the total AUDIT (*r*=–0.15, *P*=.04). No other metrics were associated with behavioral outcomes on either BART version.

### SST

#### Internal Metrics

**Figure 3:**
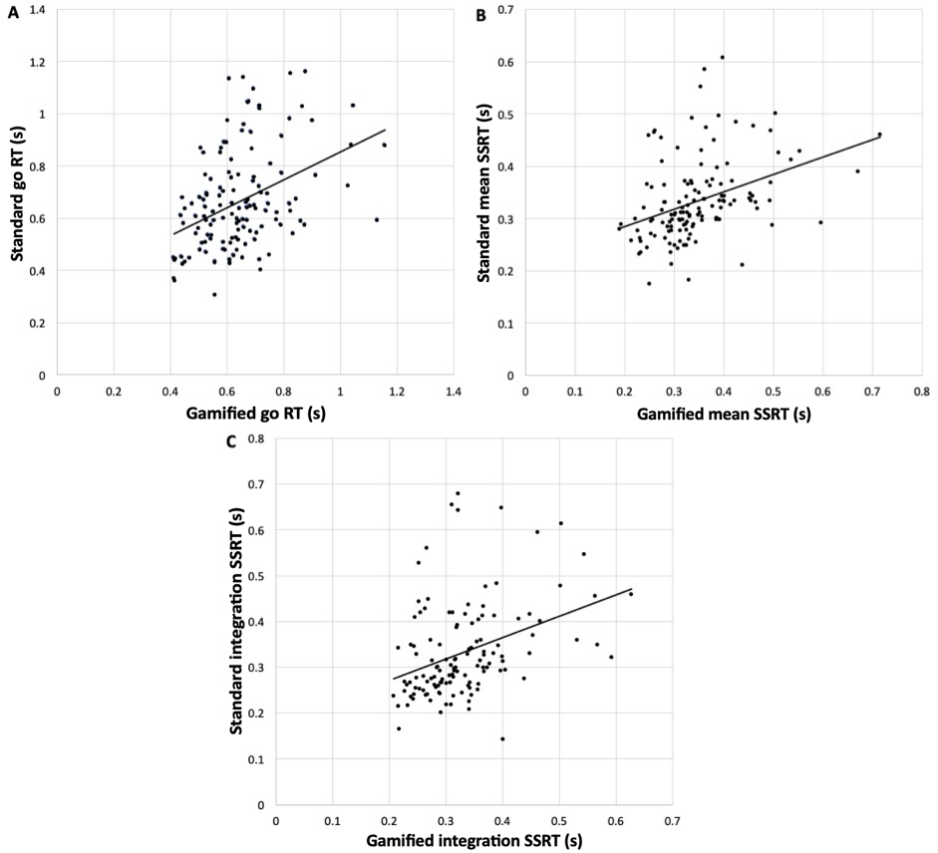
Bivariate correlations between the gamified and standard Stop Signal Task (SST) paradigms on (A) Go RT, (B) mean SSRT, and (C) integration SSRT. Go RT: go reaction time; SSRT: stop signal reaction time.

Go RT, mean SSRT, and integration SSRT all did not differ between gamified and standard SST (*P*>.05). All 3 indices were significantly associated with one another (see Figure 3).

- Go RT: *r*=0.40, *P*<.001
- Mean SSRT: *r*=0.37, *P*<.001
- Integration SSRT: *r*=0.37, *P*=.001

#### Trait Associations

No SSRT score on the gamified or standard SST was associated with trait-based scales (*P*>.05).

#### Behavioral Correlates

The mean SSRT was significantly and positively associated with ICBC impulsive behaviors on the gamified SST (*r*=0.17, *P*<.05). No other SSRT score on the gamified or standard SST was associated with other behavioral measures.

### DDT

#### Internal Metrics

The *k* function was significantly steeper in the gamified DDT than the standard DDT (*F*_1,174_=39.47, *P*<.001). On average, individuals selected the smaller sooner reward more often on the standard MCQ (59.64%) than in the gamified DDT (44.04%; *F*_1,174_=33.22, *P*<.001). The *k* function in the gamified DDT was not significantly associated with the k function on the MCQ. However, total coins earned and proportion of smaller, sooner responses on the gamified DDT were associated with the *k* function on the MCQ (*r*=–0.16, *P*=.04; *r*=0.15, *P*=.05). No other gamified and standard metrics were significantly associated with one another (*P*>.05).

#### Trait Associations

The MCQ *k* function and proportion of smaller soon responses on the gamified DDT were both associated with SUPPS-P lack of perseveration (*r*=–0.16, *P*=.04; *r*=–0.22, *P*=.004). No other trait associations were significant (*P*>.05).

#### Behavioral Correlates

The *k* function in the gamified DDT was associated with the AUDIT score (τ=0.12, *P*=.03). By comparison, the MCQ *k* function was associated with ICBC compulsive behaviors (*r*=0.19, *P*=.02), and no other behavioral measures were significantly associated with any other DDT or MCQ metrics.

### VMAC Task

#### Internal Metrics

**Figure 4:**
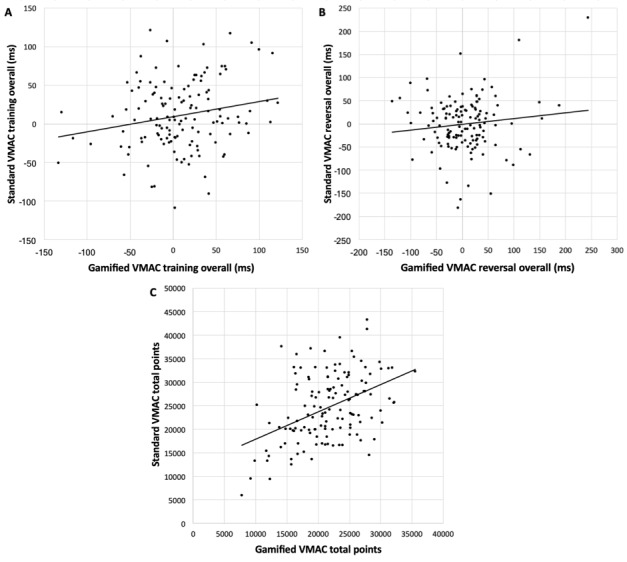
Bivariate correlations between the gamified and standard VMAC paradigms on (A) training overall, (B) reversal overall, and (C) total points. VMAC: Value-Modulated Attentional Capture.

VMAC total points were significantly higher in the standard VMAC than in the gamified VMAC; *F*_1,153_=26.98, *P*<.001). The VMAC training score and VMAC reversal score did not differ between gamified and standard variants (*P*>.05). All VMAC indices were either significantly associated with one another or approaching significance (see Figure 4).

- VMAC training overall: *r*=0.18, *P*=.04
- VMAC reversal overall: *r*=0.12, *P*=.17
- VMAC total points: *r*=0.44, *P*<.001

#### Trait Associations

The VMAC training score on the gamified task was associated with positive and negative urgency subscales on the SUPPS-P (*r*=–0.16, *P*=.045). All other associations were nonsignificant.

#### Behavioral Correlates

The VMAC reversal score on the gamified VMAC was significantly and negatively associated with ICBC compulsive behaviors (*r*=–0.20, *P*=.01), even after controlling for learning in the final training block in a bootstrapped linear regression (B=32.90, *P*=.04). No other VMAC scores on either the gamified or standard paradigms were associated with other behavioral scales.

### SDT

#### Internal Metrics

Consistent with Kool et al [59-61], mixing weight (*w*)—or greater model-based decision-making—was significantly associated with a greater total score on the gamified SDT (*r*=0.32, *P*<.001). This validates the Kool versions’ original assumptions that modifying the task would lead to model-based decision-making becoming more adaptive and advantageous to decision-making (cf. Daw version).

#### Trait Associations

(*w*) on the gamified SDT was associated with negative urgency (*r*=0.16, *P*=.03). No other trait associations were significant.

#### Behavioral Correlates

Most SDT metrics were associated with ICBC compulsive and impulsive behaviors, as follows:

- SDT inverse temperature and ICBC compulsions: *r*=0.29, *P*<.001
- SDT inverse temperature and ICBC impulsions: *r*=0.28, *P*<.001
- SDT learning rate and ICBC compulsions: *r*=–0.30, *P*<.001
- SDT learning rate and ICBC impulsions: *r*=–0.27, *P*<.001
- SDT weight parameter and ICBC compulsions: *r*=–0.30, *P*<.001
- SDT weight parameter and ICBC impulsions: *r*=–0.26, *P*<.001
- SDT total points and ICBC compulsions: *r*=–0.21, *P*=.004
- SDT total points and ICBC compulsions: *r*=–0.23, *P*=.002

No other gamified SDT metrics were associated with other behavioral scales.

### Test-Retest Reliability

The 3-day test-retest for the gamified tasks in a separate Mechanical Turk sample (n=43) was adequate for SST (integration SSRT; ICC=0.72, *P*<.001), good for DDT (*k* function; ICC=0.77, *P*<.001) and SDT (learning rate; ICC=0.86, *P*<.001), and excellent for VMAC (total points; ICC=0.91, *P*<.001). By contrast, test-retest reliability for BART (mean pumps) and the remaining VMAC and SDT indices were poor and nonsignificant (*P*>.05).

## Discussion

### Principal Findings

Two overall findings underscore the validity of the gamified BrainPAC tasks. First, most psychometric outputs (except the DDT k function and VMAC total points) did not differ between the gamified and original paradigms. Second, the small-to-large associations amongst key output metrics between these versions suggested that the gamified BART, SST, VMAC, and SDT appear to be convergent with established measures, albeit with a smaller effect than hypothesized. In keeping with the existing literature, the gamified versions of the learning paradigms VMAC and SDT were weakly associated with impulsive traits or not at all (BART, SST, and DDT), underscoring the need to measure neurocognition in addition to trait risks. Although the gamified DDT was not significantly associated with the MCQ questionnaire, similar to the gamified BART, it was sensitive to alcohol use problems. Further, the experiential version of our gamified temporal discounting task was significantly distinct from the established, nongamified hypothetical delay discounting, with each being associated with unique outcomes, suggesting they are measuring distinct constructs or aspects thereof. Interestingly, however, it appears that the gamified paradigms have stronger correlations with real-world compulsive and impulsive behaviors than the nongamified paradigms. Specifically, BART and VMAC were significantly associated with compulsive behaviors, whereas BART and SST were associated with impulsive behaviors and may reflect enhanced sensitivity to real-world actions.

In addition, some key metrics from the BART, SST, and DDT demonstrated adequate to excellent test-retest reliability and suggest that they are not only reliable measures but appropriate for risk monitoring over time. Although it stands to be replicated, the lack of test-retest for the VMAC and VMAC-R scores may be an artifact of the compounding of error variability as a result of computing difference scores [69]. Separately, the lack of test-retest for the SDT is not surprising given that, of the 5 paradigms chosen, the SDT is the only task where this has not been established in the original nongamified paradigm and warrants further study.

### Comparison to Prior Work and Implications

Very few prior studies have examined the validity and reliability of a gamified approach to cognitive assessment with the purpose of developing a comprehensive battery targeting a broad range of addictive behaviors. To our knowledge, this is the first time a comprehensive, gamified battery has been developed for the neurocognitive assessment of addictive behaviors (see Verdejo-Garcia et al [70] for a gamified battery focusing on impulsivity). The validity and reliability of the gamified tasks are comparable to those in prior studies that have adapted similar cognitive neuroscience paradigms [71-76]. However, whereas past gamification studies primarily looked at demographic associations, this study investigated convergent validity, potential predictive validity with clinical and behavioral outcomes, as well as test-retest reliability. To a large extent, our gamified tasks appear to suggest that the BrainPAC measures are sufficiently valid for use in cognitive assessment, even in the context of web-based testing, and suggest that techniques that putatively enhance engagement may have the potential to yield meaningful assessment outcomes in unsupervised testing. Whether this is due to enhanced engagement warrants specific testing that explicitly measures motivation, as well as a comparison of supervised versus unsupervised test conditions.

The current findings have significant implications for how the addiction field can articulate the current armamentarium of cognitive tools. Most tasks gamified here were, in their original forms, either well-validated by decades of research (ie, SST, BART, and DDT) or amassed a consistent empirical consensus from various research groups (ie, VMAC and SDT). Despite the existence of these individual laboratory tools, a comprehensive battery of these tasks has not been developed, validated, or made available. Moreover, there are currently no readily available versions of these tasks that are developed for use unsupervised and in a gamified format.

Current findings also suggest that our gamified DDT needs revision. The steeper discounting observed on the MCQ compared to the gamified DDT indicates that the experiential aspect of our paradigm did not achieve the same magnitude of delay as in the hypothetical paradigm. That is, people were more likely to discount rewards in the questionnaire than in the gamified task because the “cost” of time was greater. That is, we were unable to sufficiently mimic the disincentivizing nature of “waiting for larger rewards” in the short time span afforded to us by a brief neurocognitive task. Ongoing task development efforts will benefit from examining the effects of administering gamified tasks for brief periods over a number of days and embedding an experiential discounting paradigm across a longer, and perhaps more ecologically valid, timespan.

As expected, the less a paradigm is gamified (eg, BART), the greater the magnitude of association between the gamified and original variants. Whereas, when the gamified task was a significant departure from the original paradigm—as was the case for the gamified DDT, which was an experiential task rather than a hypothetical task as in the MCQ—the 2 tasks were not significantly associated. Despite this reduction in correlation, the gamified DDT remained sensitive to behavioral outcomes (ie, alcohol use), suggesting that experiential discounting tasks are likely to have unique predictive power relevant to real-world behavior. Nevertheless, the correspondence between the gamified and nongamified tasks was a little less than would be expected, and, as such, the further development of the BrainPAC tasks will need to focus on improving this association. It should be acknowledged, however, that a close-to-perfect correlation is not expected given the additional gamified elements that, as a necessity, introduce other cognitive demands.

### Limitations and Future Directions

First, given considerations regarding the length of assessment and fatigue, we did not include an in-depth suite of behavioral measures (eg, other substance use), which would have permitted a more comprehensive external validation of the BrainPAC tasks. Second, we were also unable to include all gamified tasks within a single sample due to constraints around having to already administer both versions of every task to the same participants. Third, the identified associations between measures may have been inflated due to shared measurement variance between the gamified and nongamified tasks (rather than substantive construct-related variance), as well as type I error rates (due to the number of comparisons). As such, these findings stand to be replicated. Fourth, sample characteristics differed slightly according to the time point of data collection, as may be expected (eg, due to the COVID pandemic). Future studies should also consider recruiting larger, more population-representative samples for use as normative comparisons.

In the development of the BrainPAC gamified tasks, we consulted with a cohort of users in the general community in the form of focus groups and qualitative interviews to assist us in developing tasks that individuals would find engaging. However, for the process to be maximally informed by diverse stakeholders, future refinement and iteration of the BrainPAC tasks would require further, more extensive, and continual consultation with key users and stakeholders (eg, clinical groups, community cohorts, clinicians) to further refine the BrainPAC tool as an engaging mHealth tool that users would actively engage with in the testing and monitoring of addiction risk and relapse, as well as neurocognitive functions that impact on those outcomes.

### Conclusions

On balance, the digital, gamified assessment of neurocognitive risk factors for addiction appears to be a feasible and empirically supported approach that has the potential for use in scalable mHealth tools. Critically, the psychometrics of BrainPAC tasks appear to be comparable to the corresponding nongamified assessment paradigms. It stands to be demonstrated how more sophisticated modeling in larger cohorts with more comprehensive measures of behavior can delineate how we can best measure the underlying constructs, further optimize the gamified tasks, and provide useful assessment and monitoring of brain and mental health outcomes in clinical and other relevant settings.

## Appendix

Multimedia Appendix 1 Further information on the Sequential Decision-Making Task.

Multimedia Appendix 2 Means of sample characteristics and bivariate correlation tables for the cognitive variables.

## Abbreviations

AUDIT: Alcohol Use Disorders Identification Test
BART: Balloon Analogue Risk Task
BrainPAC: BrainPark Assessment of Cognition
CHI-T: Cambridge-Chicago Compulsivity Trait Scale
DDT: Delay Discounting Task
Go RT: go reaction time
ICBC: Impulsive-Compulsive Behaviors Checklist
ICC: intraclass correlation coefficient
K10: Kessler 10-Item Psychological Distress Scale
MCQ: monetary choice questionnaire
mHealth: mobile health
RDoC: research domain criteria
SDT: Sequential Decision-Making Task
SSD: stop signal delay
SSRT: stop signal reaction time
SST: Stop Signal Task
SUPPS-P: Short UPPS-P Impulsivity Scale
VMAC: Value-Modulated Attentional Capture
