# Supplementary material for: A Novel, Expert-Endorsed, Neurocognitive Digital Assessment Tool for Addictive Disorders: Development and Validation Study
Source: J Med Internet Res. 2023 Aug 25;25:e44414. doi: 10.2196/44414 (PMC7615064; doi:10.2196/44414)
Supplement: Multimedia Appendix 1 [file jmir_v25i1e44414_app1.docx]

MEASURES

Sequential Decision-Making Task

The traditional version of the task consists of two stages with a probabilistic transition between the two. In the first stage, the participant has a choice between two stimuli, knowing that each of these stimuli will have a common (70%) or rare (30%) transition to the second stage. That is, if selecting the stimulus on the right, on 70% of the trials it will lead to second stage *a*, and on 30% of the trials to second stage *b*. In the second stage, the participants will again have a choice between two stimuli which have a probabilistic chance of leading to a reward. These reward outcomes gradually drift according to a Gaussian walk. Participants with a preference for model-free decisions will be more likely to switch after not receiving a reward, even if this occurred after a rare transition between the two stages. A model-based participant, however, would internalise the structure of the task and recognise that even though they did not receive a reward on this trial, the response they had just made was still the most strategically advantageous one as it was the most likely one to elicit rewards. A model-based agent would be able to identify the optimal strategy and apply it even after non-reward outcomes (as they would correctly attribute these to simply being bad luck rather than a strategically bad choice), whereas a model-free agent would just stick with their rewarded responses and switch after a non-reward. By analysing the stay probability following rewarded and unrewarded common and rare transition trials, Daw and colleagues [55] showed that most people used a hybrid of both model-free and model-based strategies. By fitting the data to models using both model-free and model-based RL algorithms, a weighted combination of each model using a weighting parameter could be computed (*w*). Although some participants were completely model-free (*w* = 0) and some completely model-based (*w* = 1), most relied on a hybrid of the two.

New versions of the SDT have been proposed to address theoretical concerns regarding the original paradigm [58, 59], most notably the Kool version [59, 60]. This new version does not include a common and rare transition between the two stages, but instead completely deterministic transitions. The Kool version improves on the original version of the SDT. Specifically, reward outcomes increase with model-based choices, incentivising model-based responding. The task also has a simpler structure which means maintaining a model-based strategy throughout is less demanding. Tracking the reward rate in the second stage is also easier, as the standard deviation of the Gaussian walk has been increased, making the incremental changes more noticeable. This lowers the risk of fatigue and working memory constraints. Importantly, results from studies employing this paradigm show that it still manages to elicit the full spectrum of model-free and model-based behaviour [59-61].
