# Supplementary material for: A Novel, Expert-Endorsed, Neurocognitive Digital Assessment Tool for Addictive Disorders: Development and Validation Study
Source: J Med Internet Res. 2023 Aug 25;25:e44414. doi: 10.2196/44414 (PMC7615064; doi:10.2196/44414)
Supplement: Multimedia Appendix 2 [file jmir_v25i1e44414_app2.docx]

Supplementary Table 1: Means and standard deviations of trait,

behavioural and clinical measures

|  | **M** | **SD** |
| --- | --- | --- |
| *SUPPS-P Negative Urgency* | 9.24 | 3.5 |
| *SUPPS-P Positive Urgency* | 8.81 | 4.0 |
| *SUPPS-P Lack of Premeditation* | 8.79 | 3.7 |
| *SUPPS-P Lack of Perseverence* | 8.78 | 3.5 |
| *SUPPS-P Sensation seeking* | 10.18 | 3.5 |
| *CHI-T* | 31.10 | 9.5 |
| *ICBC Impulsive behaviors* | 18.98 | 6.5 |
| *ICBC Compulsive behaviors* | 25.77 | 9.9 |
| *AUDIT* | 3.98 | 5.3 |
| *K-10* | 18.39 | 8.6 |

Supplementary Table 2: Bivariate correlations (Pearson’s) between gamified and non-gamified BART metrics

|  | Gamified BART Bursts | Gamified BART Mean pumps | Gamified BART Total money earned | Gamified BART CoV | Non--Gamified BART Bursts | Non-Gamified BART Mean pumps | Non-Gamified BART Total money earned | Non-Gamified BART CoV |
| --- | --- | --- | --- | --- | --- | --- | --- | --- |
| Gamified BART Bursts | 1 |  |  |  |  |  |  |  |
| Gamified BART Mean pumps | .89*** | 1 |  |  |  |  |  |  |
| Gamified BART Total money earned | -.91*** | -.73*** | 1 |  |  |  |  |  |
| Gamified BART CoV | -.25*** | -.32*** | .08 | 1 |  |  |  |  |
| Non--Gamified BART Bursts | .50*** | .55*** | -.41*** | -.12 | 1 |  |  |  |
| Non-Gamified BART Mean pumps | .54*** | .58*** | .42*** | -.17* | .88*** | 1 |  |  |
| Non-Gamified BART Total money earned | -.38*** | -.42*** | .34*** | .01 | -.89*** | -.68*** | 1 |  |
| Non-Gamified BART CoV | -.22*** | -.28*** | .09 | .63*** | -.20** | -.29*** | .01 | 1 |

**P*< .05; ***P*<.01; ****P*<0.001

Supplementary Table 3: Bivariate correlations (Pearson’s) between gamified and non-gamified SST metrics

|  | Gamified Go RT | Gamified Mean SSRT | Gamified Integration SSRT | Non-Gamified Go RT | Non-Gamified Mean SSRT | Non-Gamified Integration SSRT |
| --- | --- | --- | --- | --- | --- | --- |
| Gamified Go RT | 1 |  |  |  |  |  |
| Gamified Mean SSRT | .49*** | 1 |  |  |  |  |
| Gamified Integration SSRT | .79*** | .72*** | 1 |  |  |  |
| Non-Gamified Go RT | .40*** | .19* | .29*** | 1 |  |  |
| Non-Gamified Mean SSRT | .30*** | .37*** | .28*** | .66*** | 1 |  |
| Non-Gamified Integration SSRT | .36*** | .22* | .37** | .85*** | .83*** | 1 |

**P*< .05; ***P*<.01; ****P*<0.001

Supplementary Table 4: Bivariate correlations (Pearson’s) between gamified and non-gamified VMAC metrics

|  | Gamified VMAC Training overall | Gamified VMAC Reversal overall | Gamified VMAC Total points | Non-Gamified VMAC Training overall | Non-Gamified VMAC Reversal overall | Non-Gamified VMAC Total points |
| --- | --- | --- | --- | --- | --- | --- |
| Gamified VMAC Training overall | 1 |  |  |  |  |  |
| Gamified VMAC Reversal overall | .1 | 1 |  |  |  |  |
| Gamified VMAC Total points | -.15 | .05 | 1 |  |  |  |
| Non-Gamified VMAC Training overall | .18* | -.01 | .05 | 1 |  |  |
| Non-Gamified VMAC Reversal overall | .13 | .12 | -.14 | .13 | 1 |  |
| Non-Gamified VMAC Total points | -.15 | -.2 | .44*** | -.20* | -.19* | 1 |

**P*< .05; ***P*<.01; ****P*<0.001

Supplementary Table 5: Bivariate correlations (Pearson’s) between gamified SDT metrics

|  | SDT Inverse temperature | SDT Learning rate | SDT Weight parameter | SDT Total points |
| --- | --- | --- | --- | --- |
| SDT Inverse temperature | 1 |  |  |  |
| SDT Learning rate | -.85*** | 1 |  |  |
| SDT Weight parameter | -.36*** | .32*** | 1 |  |
| SDT Total points | -.32*** | .38*** | .32*** | 1 |

**P*< .05; ***P*<.01; ****P*<0.001
